# Epithelial wound healing in *Clytia hemisphaerica* provides insights into extracellular ATP signaling mechanisms and P2XR evolution

**DOI:** 10.1038/s41598-023-45424-5

**Published:** 2023-11-01

**Authors:** Elizabeth E. L. Lee, Isabel O’Malley-Krohn, Eric Edsinger, Stephanie Wu, Jocelyn Malamy

**Affiliations:** 1 Department of Molecular Genetics and Cell Biology, The University of Chicago, 929 East 57th Street, Chicago, IL 60637 USA; 2 Biological Sciences Collegiate Division, The University of Chicago, 929 East 57th Street, Chicago, IL 60637 USA; 3 Whitney Laboratory for Marine Biosciences, University of Florida, 9505 N Ocean Shore Blvd, St. Augustine, FL 32080 USA

**Keywords:** Cell biology, Evolution

## Abstract

Epithelial wound healing involves the collective responses of many cells, including those at the wound margin (marginal cells) and those that lack direct contact with the wound (submarginal cells). How these responses are induced and coordinated to produce rapid, efficient wound healing remains poorly understood. Extracellular ATP (eATP) is implicated as a signal in epithelial wound healing in vertebrates. However, the role of eATP in wound healing in vivo and the cellular responses to eATP are unclear. Almost nothing is known about eATP signaling in non-bilaterian metazoans (*Cnidaria, Ctenophora, Placozoa,* and *Porifera*). Here, we show that eATP promotes closure of epithelial wounds in vivo in the cnidarian *Clytia hemisphaerica* (Clytia) indicating that eATP signaling is an evolutionarily ancient strategy in wound healing. Furthermore, eATP increases F-actin accumulation at the edges of submarginal cells. In Clytia, this indicates eATP is involved in coordinating cellular responses during wound healing, acting in part by promoting actin remodeling in cells at a distance from the wound. We also present evidence that eATP activates a cation channel in Clytia epithelial cells. This implies that the eATP signal is transduced through a P2X receptor (P2XR). Phylogenetic analyses identified four Clytia P2XR homologs and revealed two deeply divergent major branches in P2XR evolution, necessitating revision of current models. Interestingly, simple organisms such as cellular slime mold appear exclusively on one branch, bilaterians are found exclusively on the other, and many non-bilaterian metazoans, including Clytia, have P2XR sequences from both branches. Together, these results re-draw the P2XR evolutionary tree, provide new insights into the origin of eATP signaling in wound healing, and demonstrate that the cytoskeleton of submarginal cells is a target of eATP signaling.

## Introduction

Sheets of connected epithelial cells cover the surfaces of all animals and line internal cavities and organs. Epithelial sheets represent a critical step in metazoan evolution as they divide animal bodies into discrete compartments. They are also essential for protecting tissues from external insults and infectious agents^1^. Epithelial sheets inevitably sustain damage which must be rapidly repaired. Epithelial wound healing has been studied in a wide variety of adult and embryonic animal models. In the embryos of mouse, *Drosophila*, chick, *Xenopus* and other organisms, epithelial wound healing is accomplished through a combination of lamellipodia-based crawling and/or the contraction of a multicellular actomyosin cable that draws the cells at the wound margin together in a purse-string mechanism^2–6^. These mechanisms also drive closure of small wounds in adult vertebrate tissues such as the intestinal and corneal epithelium^3,4,7,8^ and in cutaneous wounds in zebrafish^9^. In larger epithelial wounds in adult vertebrate tissues, monolayers of epithelial cells in culture, and *Drosophila* larvae, marginal cells and cells at a distance from the wound (submarginal cells) migrate together to close gaps, moving either as coherent sheets or as groups of independent cells that move collectively into the wound region^10–15^, depending on the tissue examined^16,17^. Much remains to be learned about the mechanisms that control these processes.

Relatively little work has been done on wound healing in the non-bilaterian metazoans such as cnidarians^18–20^. Nevertheless, these organisms can reveal the evolutionary origin of wound healing mechanisms and likely identify mechanisms that are conserved across the tree of life. Furthermore, non-bilaterian metazoans provide a unique opportunity to isolate and study core elements of epithelial healing in vivo as they have a simplified architecture and lack the concurrent processes that occur in more complex animals (i.e. release of vascular factors, inflammation and fibroblast recruitment).

**Figure 1:**
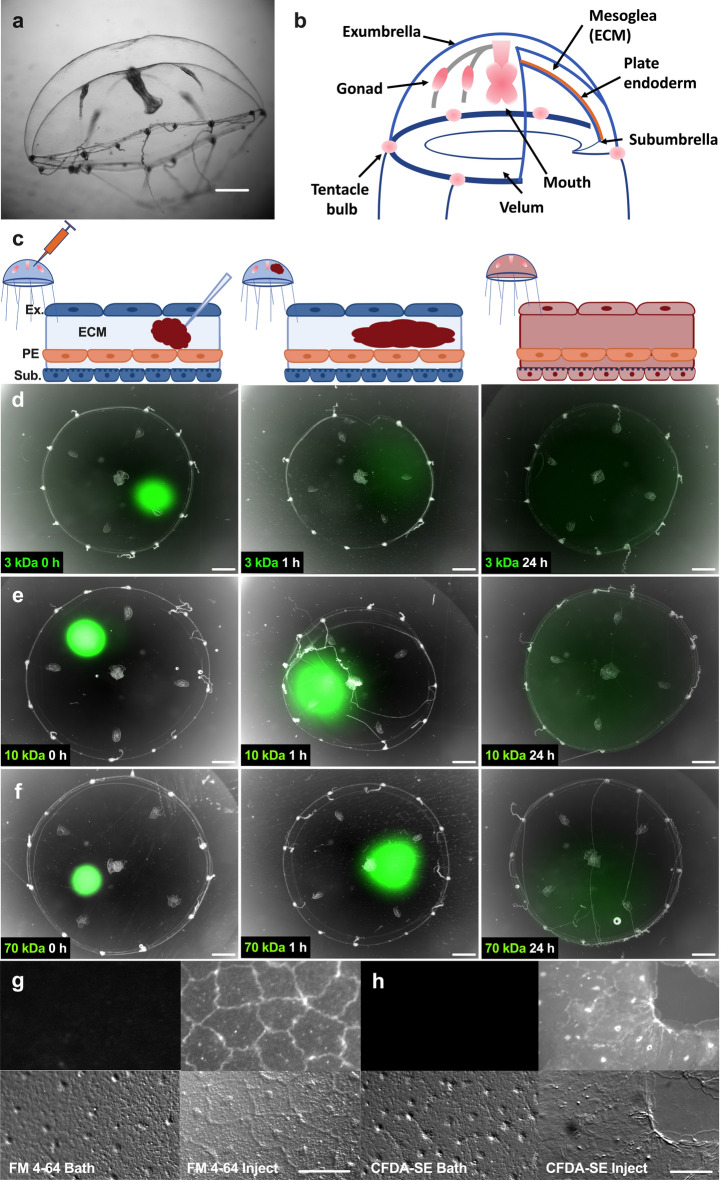
Microinjection permits drugs and dyes to access the ECM and epithelial cell cytoplasm. (**a**) Side view of an adult Clytia medusa, scale bar = 3 mm. (**b**) Schematic representation of *Clytia* medusa. (**c**) Schematic representation of microinjected reagents diffusing in the mesoglea (ECM). Image created with Biorender.com. (**d**–**f**) Time course of the diffusion of 100 µM neutral-charge fluorescent-labeled dextrans at 0 (left), 1 (center), and 24 (right) hours post injection. (**d**) 3 kDa (**e**) 10 kDa (**f**) 70 kDa. Scale bars = 1 mm. Apparent variations in medusa sizes are due to movement of the animal during imaging. (**g–h**) Imaging of fluorescent dyes in the exumbrella epithelium (upper panel) and the corresponding site with DIC microscopy (lower panel). (**g**) 10 µM FM 4–64 dye, 1 h after bath application (left) or 5 min after microinjection (right). (**h**) 100 µM CFDA-SE dye, 1 h after bath application (left) or 15 min after microinjection (right). Scale bars = 50 µm. Abbreviations: ECM: extracellular matrix, Ex: exumbrella, Sub: subumbrella, PE: plate endoderm.

We recently introduced the cnidarian *Clytia hemisphaerica* (Clytia) as a compelling model for studying epithelial wound healing*.* Clytia is a representative of an evolutionarily ancient lineage; cnidarians diverged from the bilaterian lineage over 600 mya. Therefore, signaling pathways found in vertebrates and in cnidarians were likely present in the last common ancestor. Furthermore, previous characterization of wound healing in Clytia medusae showed examples of lamellipodia-based cell crawling, cell sheet migration and purse-string contraction^21^, demonstrating that Clytia is a useful model for processes that are present in a wide range of animals. Importantly, Clytia offers the ability to image the dynamics of epithelial cell movement at high resolution in live, intact animals. The Clytia exumbrella (the upper side of the medusa bell) is made up of a monolayer of large (~ 50 µm in diameter) squamous epithelial cells that rest on the mesoglea, a transparent acellular^22^ gel, 200–500 µm thick, that it is analogous to the extracellular matrix (ECM) of other animals (Fig. 1a,b)^23–25^. Immediately below the exumbrella epithelial cells is a basement membrane^21^, a specialized region of the ECM that forms a surface and separates epithelial cells from the rest of the ECM in all multicellular animals^24,26^. Hence, anatomically the exumbrella epithelial cells of Clytia have all the characteristics of epithelial sheets in more complex animals. This epithelial monolayer can be manually damaged*,* and the movements of the epithelial cells as the wound heals can be imaged at high spatiotemporal resolution^21,27^. Another unique and critical feature of Clytia is that the ECM is large and easily accessible, allowing perturbation of the ECM and cell:ECM interactions during wound healing.

Here we use tools developed in Clytia to dissect cellular responses to small epithelial wounds in vivo, focusing on extracellular ATP (eATP) signaling. eATP is a rapid transcription-independent signal believed to play a critical role in the early stages of wound response in many organisms^28–31^. ATP is normally found at nanomolar levels in the ECM, while intracellular concentrations are estimated at around 5 millimolar^30^. Hence, the dramatic increase of eATP when cells are broken serves as a ubiquitous damage signal^28–32^. While increases in eATP have been seen in many in vivo contexts in response to cell stress or damage^28,30,32^, most studies on the downstream effects of eATP on epithelial cells have been done in vertebrate tissue culture. eATP binds to purinergic receptors of the P2XR and P2YR families in cultured vertebrate epithelial cells and is necessary and sufficient to elicit waves of intracellular Ca^+2^ in epithelial cell monolayers and ex vivo corneas^30,33–37^ and in the developing *Xenopus* brain^38^. Importantly, eATP signaling has been shown to promote cell migration and wound closure in scratch assays in vitro in epithelial cell monolayers^34,35,39–42^ and in vivo eATP was shown to promote cell migration and healing in wounded zebrafish tail fins^43^.

The present study shows that eATP released by wounding promotes epithelial wound healing in Clytia. We also identified actin remodeling as a cellular target of eATP signaling. Specifically, in Clytia, eATP promotes accumulation of actin at the edges of submarginal cells, indicating a role for this diffusible signal in coordinating responses between marginal cells and cells at a distance from the wound site. We further demonstrate the presence of ATP-gated cation channels in Clytia epithelial cells and provide pharmacological and genomic evidence consistent with these channels being P2XRs. This suggests an ancient origin of eATP signaling through P2XRs in epithelial wound healing. Phylogenomic analysis in Clytia and across eukaryotes led to a re-modeling of the P2XR phylogenetic tree, revealing two deeply divergent branches in P2XR evolution that were not previously recognized. Vertebrate P2XR genes are limited to one evolutionary branch, simple eukaryotic organisms like cellular slime mold are limited to the other, and some non-bilaterian metazoans, including Clytia, are surprisingly represented in both branches.

## Results

### Microinjection allows the efficient introduction of reagents into Clytia ECM and epithelial cells

To study epithelial wound healing in Clytia, it is critical to be able to introduce dyes and pharmacological agents to the ECM, the surface of the exumbrella epithelial cells, and the epithelial cell cytoplasm (Fig. 1a,b). Attempts to introduce reagents into *Clytia* epithelial cells by adding them to a saltwater bath surrounding a medusa were unsuccessful. For example, FM4-64 and CFDA-SE, widely used to label cell membranes and living cell cytoplasm, respectively, showed no staining when added to live *Clytia* medusa as a bath (Fig. 1g,h). We therefore investigated whether we could use microinjection into the ECM to overcome this technical challenge. Since the Clytia ECM is easily accessible, microinjection into this compartment should allow reagents to access the ECM, the basal surface of the epithelial cells and the epithelial cell cytoplasm (Fig. 1c).

To test the idea that we can introduce small molecules into the ECM without harming the animal we used a microinjection needle to inject ~ 5 uL of Fast Green dye. This treatment had no apparent detrimental effects—animals remained healthy at least 7 days after the injection. To determine how well molecules diffuse throughout the ECM, we used fluorescent neutral dextrans of varying sizes (3, 10 and 70 kDa). These molecules diffused away from the injection site within an hour, and throughout the ECM by 24 h post injection (Fig. 1d–f). Further, injection of Hoechst dye showed that reagents locally injected into the mesoglea can access cells throughout the animal (Supplementary Fig. S1). We next injected FM4-64 or CFDA-SE into the ECM. These dyes were able to gain entry to the cell membrane and cytoplasm, respectively, ostensibly from the basal side (Fig. 1g,h). This is consistent with our previous results that microinjected FM1-43 stained epithelial cells^22^. Hence, microinjection allows delivery of reagents to the ECM and to the basal surface of the exumbrella epithelial cells and overcomes previous technical problems in introducing dyes and inhibitors into epithelial cell cytoplasm in Clytia.

### Small epithelial wound healing is transcription-independent

**Figure 2:**
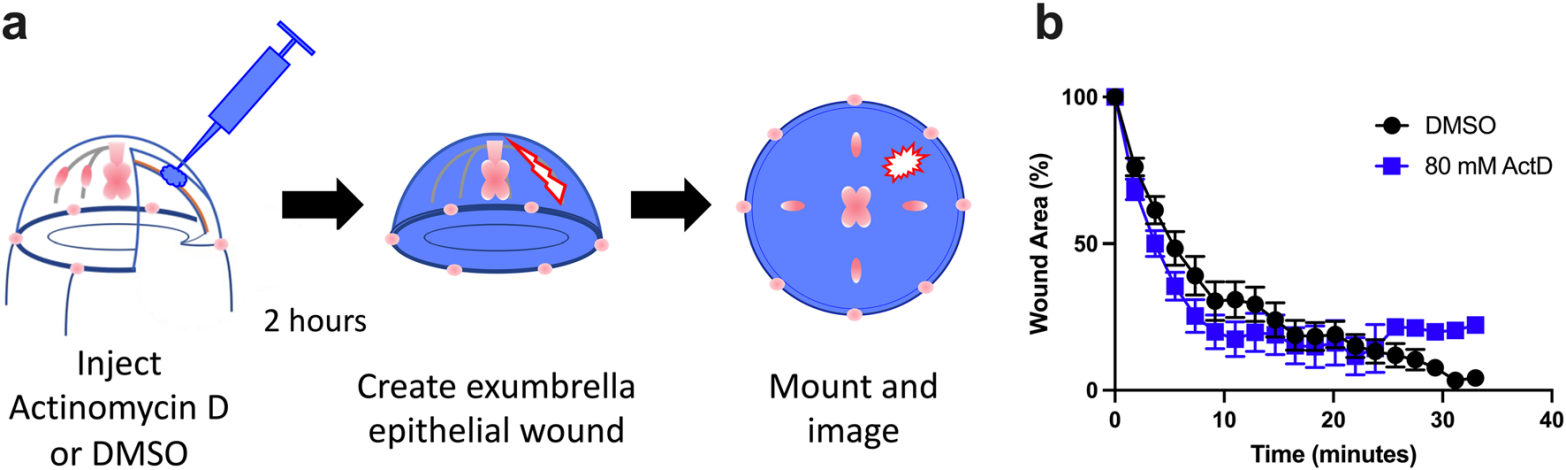
De novo transcription is not required for healing of small epithelial wounds. (**a**) Schematic representation of the experimental setup. (**b**) Inhibition of transcription by 80 mM actinomycin D (ActD) did not cause a significant difference in wound healing rate (n = 12) compared to DMSO (n = 11). n = number of wounds analyzed in independent animals. Data is presented as mean percent of original wound area ± SEM. Unpaired two-tailed T-test at each time point found *P* > 0.05.

As a first step in dissecting the cellular responses to epithelial wound healing in Clytia, we asked whether wound-induced gene expression is required. We focused on small wounds in the exumbrella epithelium (0.02–0.125 mm^2^). These wounds heal primarily through lamellipodia-based crawling of the cells at the wound margin; once lamellipodia encounter each other across the wound gap the tissue around the wound contracts, cells adhere to each other, and a scar-free epithelial sheet is restored^21^. Using the Click-iT®RNA Imaging Kit, which follows the incorporation of ethynyl uridine (EU) into RNA to reveal de novo transcription, we found that the exumbrella epithelial cells were transcriptionally active in both wounded (90.9% EU positive cells, n = 77 nuclei in two animals) and unwounded animals (86.4% EU positive cells, n = 60 nuclei in two animals). Actinomycin D (80 mM) microinjection strongly inhibited de novo transcription throughout the exumbrella epithelium in the window 2–4 h post-injection (Supplementary Fig. S2). Therefore, wounds were created 2 h after injection and the rate of wound closure was measured over the subsequent 30–40 min required for healing (Fig. 2a). No detectable change in the rate of wound closure was seen when de novo transcription was inhibited (Fig. 2b), nor did we observe any change in the initiation of wound healing events or their progression. This suggests that wound-induced gene expression is not necessary for effective healing of small wounds in Clytia.

### Extracellular ATP enhances the rate of wound closure

**Figure 3:**
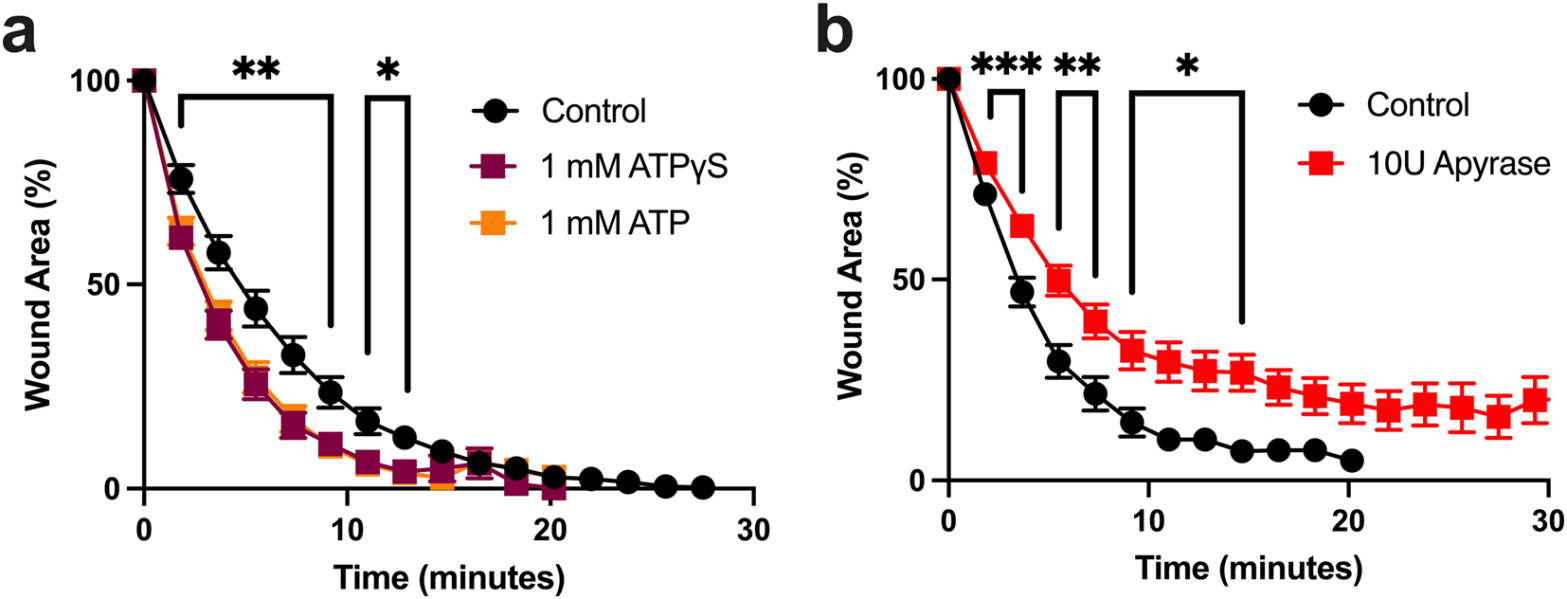
eATP promotes closure of epithelial wounds. (**a**) Microinjection of 1 mM ATP (n = 11) or 1 mM ATPγS (n = 9) 5 min before wounding increases wound closure rate in comparison to ASW-injected controls (n = 9). One-way analysis of variance (ANOVA) at each time point: *P* < 0.01 (**); *P* < 0.05 (*). (**b**) Microinjection of 10U of apyrase, an ATP scavenger (n = 22), 30–60 min before wounding slows healing rate in comparison to ASW-injected controls (n = 13). Unpaired two-tailed T-test at each time point: *P* < 0.05 (*); *P* < 0.01 (**); *P* < 0.001 (***). n = number of wounds analyzed in independent animals. Data is presented as mean percent of original wound area ± SEM.

Given the fast rate of wound healing in Clytia^21^ and the lack of effect of transcriptional inhibition, we next focused on rapid, transcription-independent signaling pathways known to promote wound healing in other systems^30,44,45^. ATP is a ubiquitous molecule in living systems, and eATP is a wound and damage signal in both plants and animals^31,44–46^. When ATP was injected into the Clytia ECM immediately prior to wounding there was a small but significant increase in the rate of wound closure (Fig. 3a). Similar results were seen with the non-hydrolyzable ATP analog ATPγS, confirming that eATP is acting as a signal rather than an energy source to promote healing (Fig. 3a). A comparable increased rate of wound closure was seen when the pH of the ATP was adjusted to 8.2 to match artificial sea water (ASW, control) eliminating the possibility of a pH effect (Supplementary Fig. S3). Hydrolyzing endogenous eATP by injecting apyrase into the ECM in advance of wounding resulted in a reduced rate of wound closure (Fig. 3b). Notably, the changes to the wound closure rate were during the first 10 min of healing. Together, these results show that eATP released by wounding promotes the early stages of the healing process.

### Wound-induced actin remodeling is promoted by eATP signaling

eATP is known to trigger increases in intracellular Ca^+2^ in response to wounding, but there is little additional information about eATP effects at the cellular level. A hallmark of epithelial wound healing is the accumulation of actin at the front of marginal cells and the formation of actin-based lamellipodia that extend into the wound gap^47–49^. Actin accumulation at cell fronts is also seen in submarginal cells^47–51^. We tested whether wound-induced changes in actin accumulation are a target for eATP signaling in Clytia.

**Figure 4:**
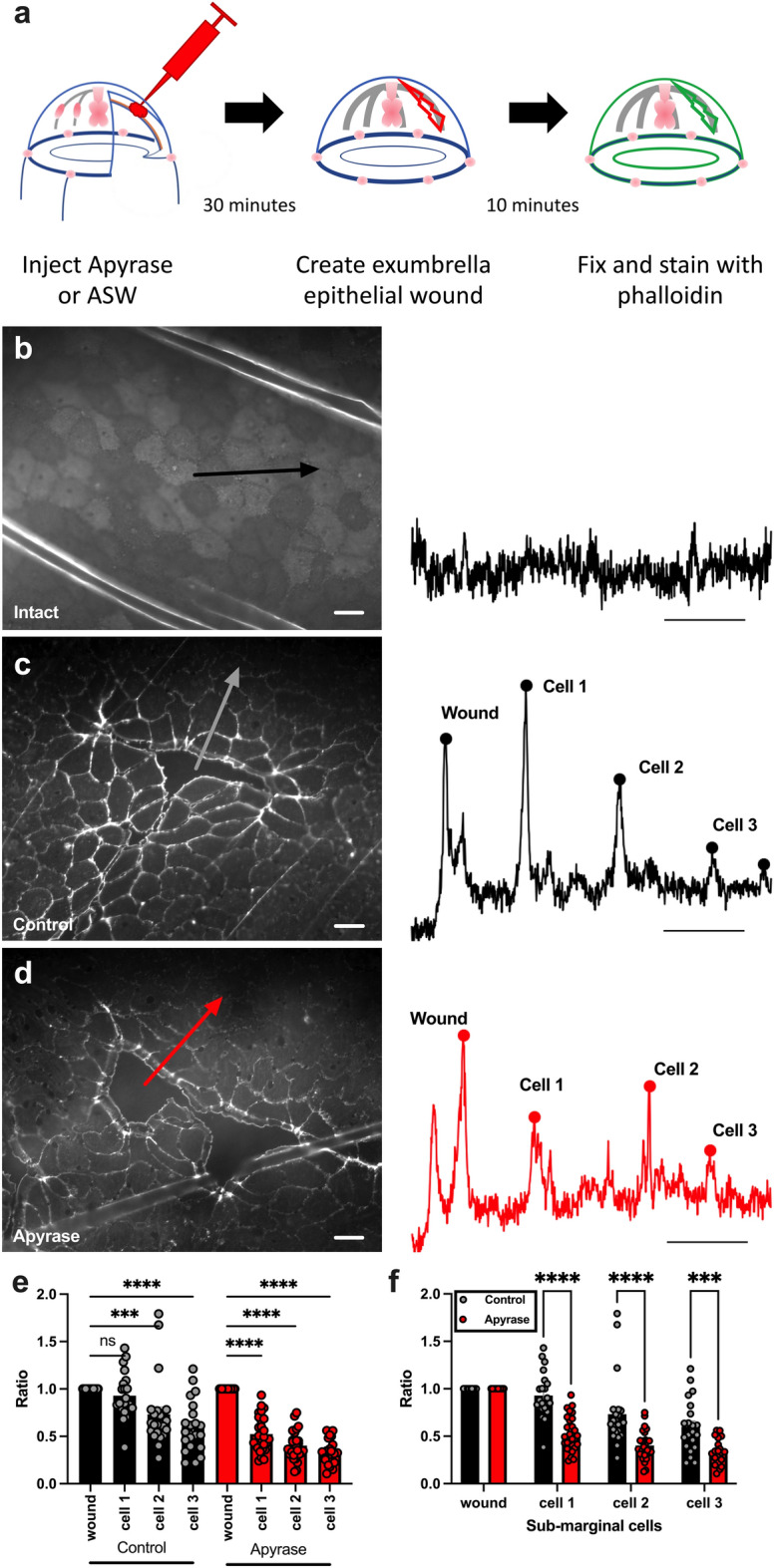
Apyrase treatment inhibits actin accumulation in epithelial wound sub-marginal cells. (**a**) Schematic representation of experimental setup. Animals were injected with 10 U of apyrase or ASW (control), wounded and then fixed for actin imaging. (**b–d**) Representative images of epithelial cells in animals after various treatments (left) and corresponding fluorescence intensity profile for the arrow in each image (right). Arrows indicate the line and direction of the example intensity profile. Scale bars = 50 µm. (**b**) Unwounded animal. (**c**) ASW-injected (control) animal. (**d**) Apyrase-injected animal. (**e, f**) Peak fluorescence intensity of actin accumulation at the wound-margin cell (wound) and sub-marginal cells (cell 1–cell 3). Fluorescence in each sub-marginal cell is normalized to the peak fluorescence of the wound-margin cell for each treatment. The same data is shown in e and f to represent distinct statistical comparisons. Data points represent mean fluorescent intensity of all wounds in an animal. For each wound, intensity profiles were determined on 4–8 lines. 1–5 wounds were measured per animal. Bars are the means of all data points ± SEM. Control: n = 22; Apyrase treated: n = 28. n = number of animals. (**e**) Sub-marginal cells accumulate less actin than wound-margin cells in both ASW-(control) and apyrase-injected animals. Ordinary one-way ANOVA with a post hoc Tukey test for multiple comparisons: *P* = 0.0005 (***); *P* < 0.0001 (****). (**f**) Sub-marginal cells from apyrase-injected animals have less actin accumulation than corresponding cells from ASW-injected animals. Unpaired two sample t-test: Cell 1, *P* < 0.000001 (****); Cell 2, *P* = 0.000088 (****); Cell 3, *P* = 0.000284 (***). ns = not significant *P* > 0.05.

Phalloidin staining showed that wounding induced a dramatic increase in actin accumulation at the wound-side of marginal cells in comparison to unwounded animals (Fig. 4a–c). Actin accumulation was also induced at the edges of submarginal cells 3–4 cells away from a wound margin, although to a lesser extent (Fig. 4c,e). To test the role of eATP in wound-induced actin remodeling, apyrase was injected into the ECM of animals before wounding. 10 min after wounding, animals were fixed, stained, and imaged for actin. Actin accumulation and lamellipodia formation at the front edge of marginal cells showed no apparent differences with or without apyrase (Fig. 4c,d). In contrast, there was a striking reduction in the accumulation of actin at the edges of submarginal cells (Fig. 4c–f). The submarginal cells are not expected to participate in the closure of the small epithelial wounds characterized in these experiments, and therefore the altered actin localization may not explain the observed reduction in the rate of wound closure in the presence of apyrase. Nevertheless, these results show that small wounds induce cellular changes at a distance from the wound, and actin remodeling in submarginal cells is a target of eATP signaling.

### eATP likely activates P2XR cationic channels in exumbrella epithelial cells

eATP binds and activates two types of unrelated purinergic receptors in animals—P2XRs and P2YRs^28^. P2XRs are trimeric ion channels that, upon ATP binding, allow extracellular cations such as Ca^2+^ to enter the cell. P2YRs, in contrast, are G-protein coupled receptors whose activation by ATP leads to the release of internal Ca^+2^ stores through inositol-3-phosphate signaling. Analysis of the Clytia genome revealed no P2YR family genes, consistent with evidence that P2YRs appeared much later in evolution^31^. Therefore, we tested the hypothesis that the eATP signal is transduced by a P2XR in Clytia epithelial cells.

**Figure 5:**
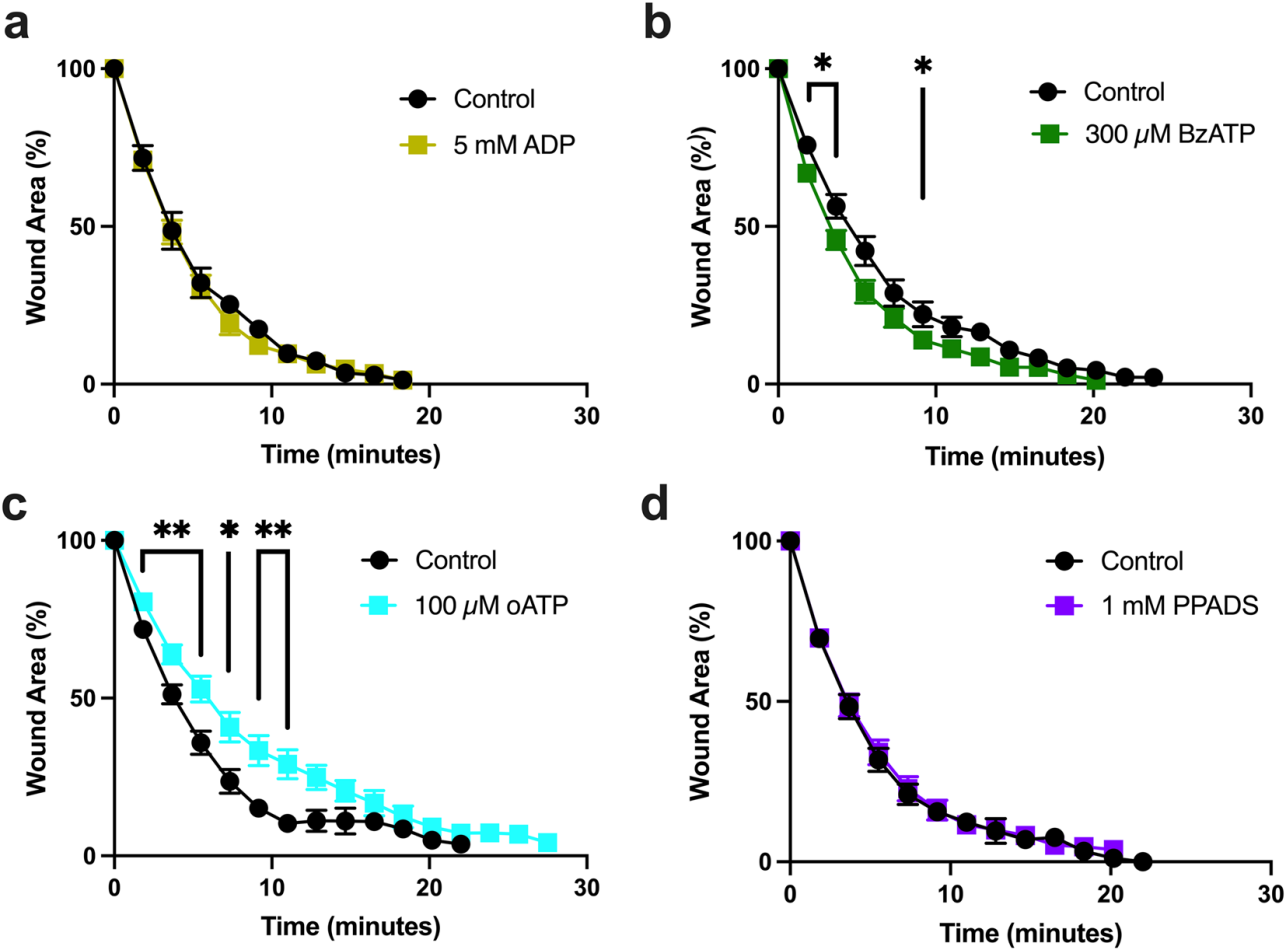
Effects of ATP receptor agonists and antagonists on epithelial would healing. (**a**) 5 mM ADP (n = 8), a P2YR agonist, has no effect on healing when injected 5 min before wounding in comparison to ASW controls (n = 8). (**b**) 300 µM BzATP (n = 16) a P2XR agonist, speeds early stages of wound healing when injected 1–3 h before wounding in comparison to ASW controls (n = 16). (**c**) 100 µM oxidized ATP (oATP, n = 16), a P2XR antagonist, slows wound healing when injected 1–3 h before wounding in comparison to ASW controls (n = 11). (**d**) 1 mM PPADS (n = 12), a general P2XR agonist, has no effect on rate of healing when injected 30 min before wounding in comparison to ASW controls (n = 13). n = number of wounds analyzed in independent animals. Data is presented as mean percentage of original wound area ± SEM. Unpaired two-tailed T-test at each time point: *P* < 0.05 (*); *P* < 0.01 (**).

We first tested the effect of ADP, which activates P2YRs but not P2XRs^52^, and found no effect on wound healing (Fig. 5a). Consistently, 2′(3′)-O-(4-benzoylbenzoyl)adenosine-5′-triphosphate (BzATP), which is a strong agonist of P2XRs in vertebrates^53^, increased the wound healing rate (Fig. 5b), while the P2XR-specific inhibitor oxidized ATP (oATP)^54^ reduced it (Fig. 5c). This pharmacological profile supports the hypothesis that the effect of eATP on wound healing is mediated by a P2XR. Interestingly, pyridoxal-phosphate-6-azophenyl-2′,4′-disulphonate (PPADS), an inhibitor of many animal P2XRs^55,56^, had no effect on wound healing (Fig. 5d). However, PPADS sensitivity is species- and gene-specific, and at least one other cnidarian P2XR has been shown to be insensitive to PPADS^57^ (see below).

**Figure 6:**
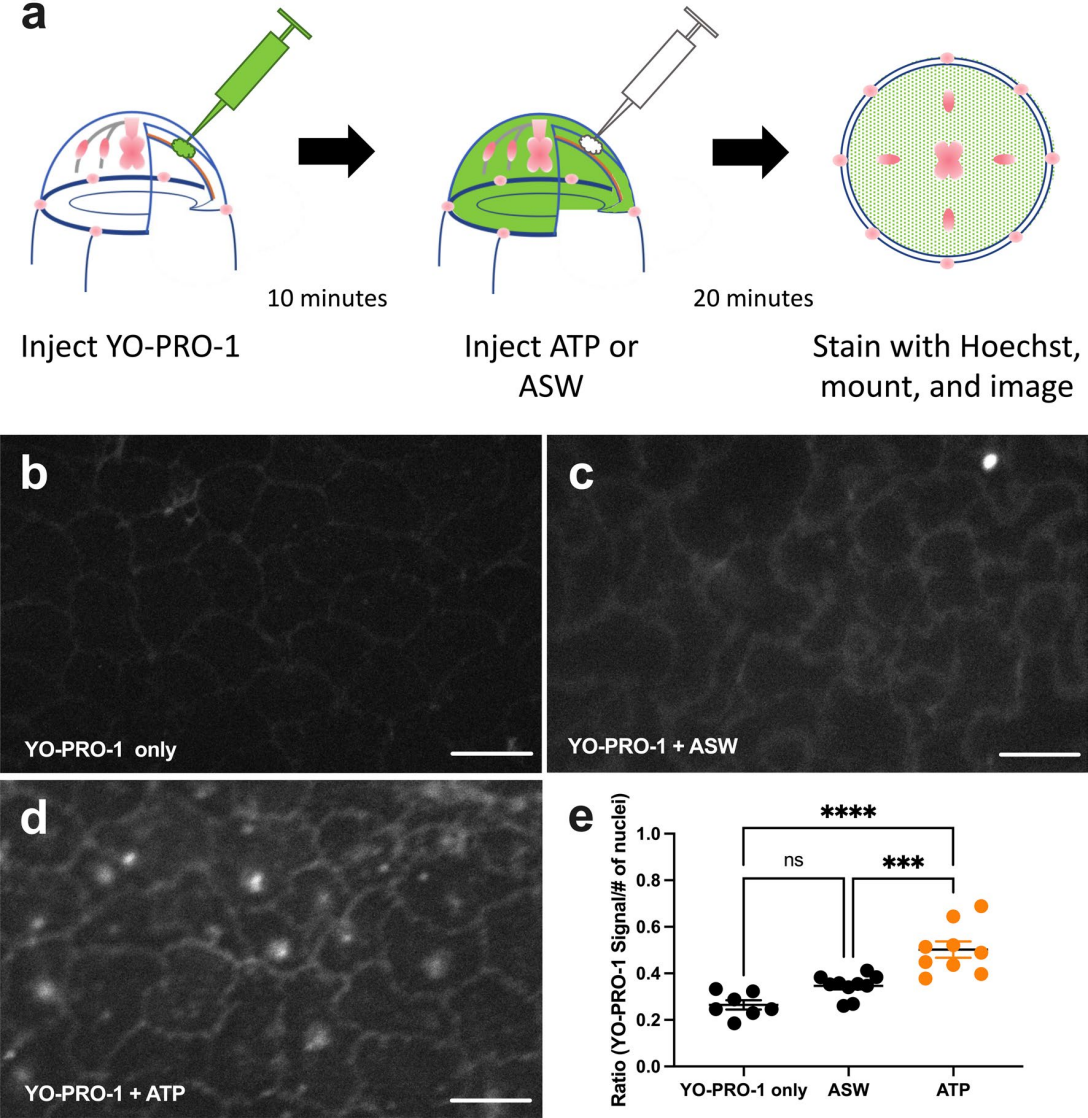
ATP injection allows YO-PRO-1 to enter exumbrella epithelial cells. (**a**) Schematic representation of the experiment. Animals were injected with YO-PRO-1, incubated for 10 min, and either left untreated or injected with 1 mM ATP or ASW. 20-min later animals were stained with Hoechst, mounted, and all four quadrants were imaged. (**b–d**) Representative images of exumbrella epithelial cells with either (**b**) YO-PRO-1 only; (**c**) YO-PRO-1 + ASW control; (**d**) YO-PRO-1 + 1 mM ATP. Scale bar = 50 μm. (**e**) Quantification of YO-PRO-signal in all four quadrants of animals treated with YO-PRO-1 only (n = 7), YO-PRO-1 + ASW (n = 10) or YO-PRO-1 + 1 mM ATP (n = 9). ATP injection significantly increased the entry of YO-PRO-1 into the epithelial cells. The number of YO-PRO-1 particles across the image were normalized by the number of nuclei. Datapoints represent the mean normalized particle number from four images per animal. n = number of animals imaged. Horizontal bars represent mean of datapoints within a treatment ± SEM. Ordinary one-way ANOVA: *P* =  < 0.0001 (****); *P* = 0.0004 (***); ns = not significant, *P* > .05.

When ATP activates P2XRs, they allow the passage of cationic dyes such as YO-PRO-1. Therefore, to detect the presence of P2XR channels in Clytia, we injected YO-PRO-1 into the ECM (Fig. 6a). Animals injected with YO-PRO-1 alone showed minimal entry of dye into the epithelial cells (Fig. 6b,e). In contrast, injection of ATP resulted in the movement of YO-PRO-1 into the epithelial cell cytoplasm and nuclei (Fig. 6d,e). (Animals injected with ASW also showed some cellular accumulation of YO-PRO-1, although it was not statistically different from YO-PRO-1-only controls (Fig. 6c,e). The injection process itself damages cells and releases eATP, which likely explains the YO-PRO-1 increase in cells in some regions of ASW-injected animals.) This shows that exumbrella epithelial cells have ATP-gated cation channels. This suggests that eATP, released into the ECM by wounding, activates P2XRs on the epithelial cell basal surface.

### Clytia has representatives of two highly divergent branches of P2XR evolution

P2XRs have been identified in the genomes of a wide variety of eukaryotes, from single-cell organisms such as green algae, choanoflagellates, and cellular slime mold to complex animals^53,58,59^. In Clytia, four transcripts were annotated as encoding P2XRs (http://marimba.obs-vlfr.fr/): TCONS_00012680; TCONS_00058261; TCONS_00058302; and TCONS_00058308.

To explore the diversity and evolution of the P2XR gene family in Clytia, over three hundred genome-based proteomes (Supplemental Table S1) were evaluated for completeness using the BUSCO Metazoa database and seventeen species were selected for genome-scale phylogenetic analysis (Supplemental Table S2). P2XR genes are well documented in human^28,29,59,60^ and in the cellular slime mold *Dictyostelium discoidea*^58,61–63^ and these genes were used to form the reference gene set. (For the human P2X7 gene, the C-terminal sequence (356–595) was trimmed in the reference gene set.  This sequence encodes the P2X7 C-terminal domain, which was likely acquired by fusion to a P2X4-like gene during evolution of fishes; there is therefore extensive similarity of these sequences with sequences in unrelated gene families^16^.) Reference genes were BLASTED against each of the seventeen target genomes, including their own. Similar to the transcriptome, we found four P2XR genes in the Clytia genome, which we designate ChP2X1 (XLOC_007123), ChP2X2 (XLOC_036701), ChP2X3 (XLOC_036723) and ChP2X4 (XLOC_036725). ChP2X2, ChP2X3 and ChP2X4 occur as a tandem array (Supplementary Table S3).

**Figure 7:**
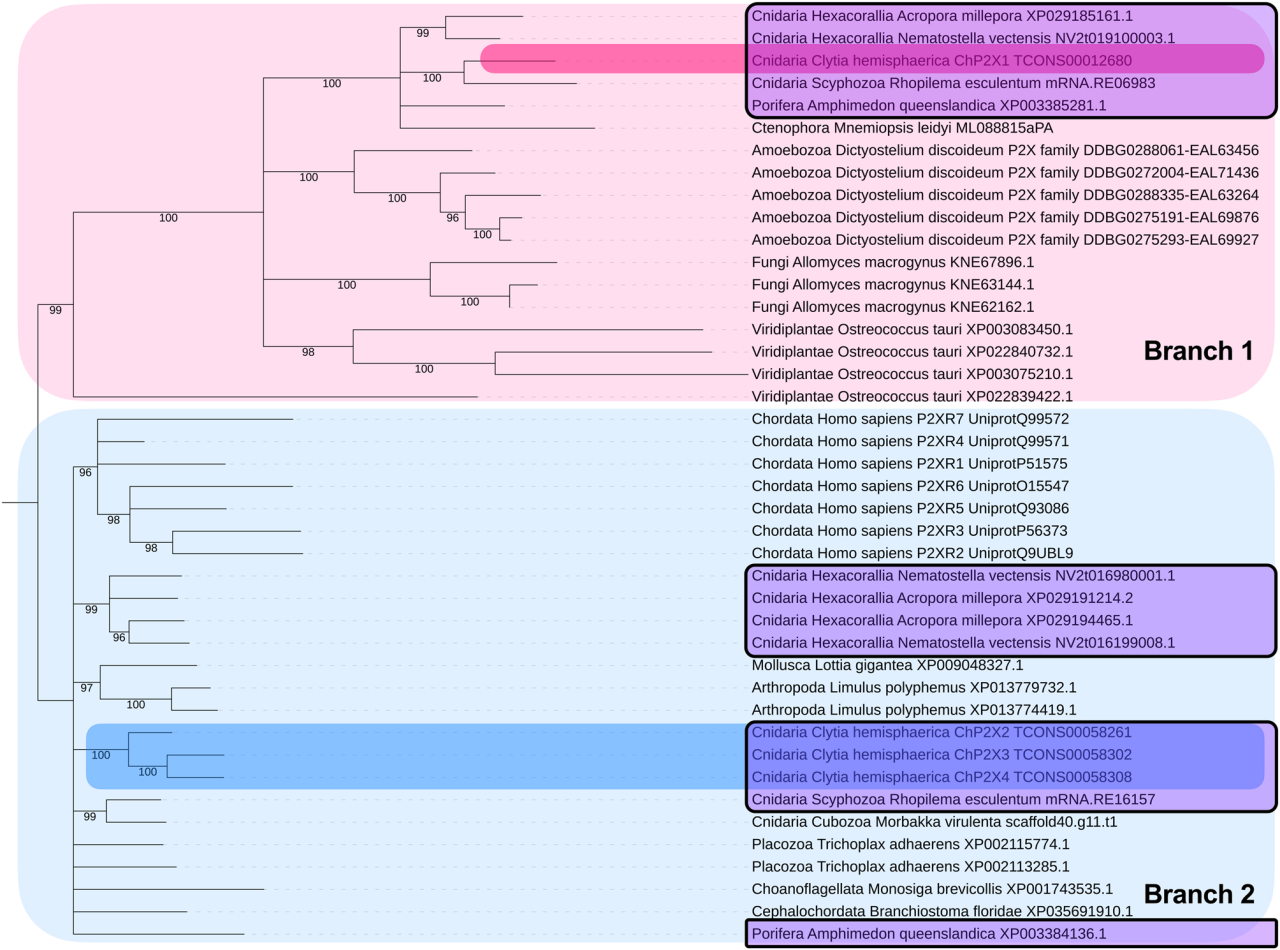
Phylogenetic tree of P2XRs across eucaryotes. Homologs were detected by reciprocal BLAST back to reference gene family. Sequences were aligned in MAFFT, trimmed in ClipKit, and maximum likelihood trees produced in IQTree (see Methods). The tree was rooted based on the deepest branch across eukaryotes. Branches having 95% or greater ultrafast bootstrap support are considered significant under ultrafast bootstrapping guidelines^91^ and were retained while branches with less than 95% ultrafast bootstrap support are considered unreliable and have been collapsed. The tree is arbitrarily rooted at the deepest node of the Clytia sequence ChP2X1 lineage having significant ultrafast bootstrap support. Representatives for Branch 1 are shown in pink and Branch 2 in blue. The four Clytia P2XR homologs are highlighted. An unrooted un-collapsed version showing all bootstrap values is presented in Supplement Fig. S3. Purple boxes highlight organisms with P2XRs in both branches.

Surprisingly, phylogenetic analysis revealed a previously unrecognized deep eukaryotic divergence in the evolution of the P2XR family that is reflected in animals, including Clytia (Fig. 7; Supplementary Fig. S4). P2XR Branch 1 is defined by P2XR diversity in *Dictyostelium* and other diverse eukaryotic lineages including green algae and fungi (Fig. 7; Supplementary Fig. S4). In animals, Branch 1 includes representatives of sponges, ctenophores, and several cnidarian classes (*Hydrozoa*, *Scyphozoa*, and *Hexacorallia*), including previously unrecognized P2XR genes in *Nematostella* and *Acropora*. Branch 1 does not have placozoans or any bilaterian representatives. In contrast, P2XR Branch 2 is defined here by human sequences, as well as commonly recognized P2XR diversity throughout animals, including non-bilaterian metazoans and diverse cnidarian classes (*Hydrozoa, Hexacorallia, Scyphozoa* and *Cubozoa*), with several examined here for the first times (Fig. 7; Supplementary Fig. S4). In sum, we find two highly divergent branches in P2XR evolution, and conclude that the Clytia genome contains both deeply conserved (ChP2X1) and more recently evolved P2XR homologs (ChP2X2-4). A range of other cnidarian species and sponge also contained sequences from both evolutionary branches (purple boxes, Fig. 7), while all other phyla examined were restricted to one branch or the other. These findings substantively change previous models for P2XR evolution^58,64,65^.

### Sequence alignments suggest that Clytia homologs from both evolutionary branches are functional P2XRs

We next aligned all Clytia P2XR protein sequences to the four *Dictyostelium* proteins experimentally shown to function as ATP-gated ion channels (Branch 1) and the well-characterized human P2X1 (Branch 2) to look for evidence of conserved functionality. All four Clytia proteins have either identical or similar amino acids at positions previously shown to be conserved in functional P2XRs across the tree of life^66^ (Supplementary Fig. S5). ChP2X2, ChP2X3 and ChP2X4 (Branch 2) proteins have high overall similarity with human P2X1 (~ 60%) and contain conserved amino acids in positions known to be critical for ATP-binding in vertebrate proteins (Lys68, Lys70, Arg292, and Lys309 in human P2X1^67^) (Supplementary Fig. S5), strongly supporting conservation of function in the Clytia Branch 2 proteins. ChP2X1 (Branch 1) lacks two of the ATP-binding amino acids conserved in Branch 2 proteins and aligns poorly overall with human P2X1 but has up to 47% amino acid identity or similarity with *Dictyostelium* proteins. Like ChP2X1, the *Dictyostelium* P2XRs lack two or more of the four Branch 2 ATP-binding amino acids^61^, yet ATP-specific currents were evoked when these proteins were expressed in heterologous systems^61–63^. This observation suggests that Branch 1 proteins, including ChP2X1, are also functional P2XRs with a distinct mechanism of ATP binding compared to proteins from Branch 2.

### Different pharmacological sensitivity defines the two P2XR branches

We also analyzed P2XR sequences associated with PPADS sensitivity in Branch 1 and 2 proteins. The specific amino acids essential for PPADS inhibition of P2XRs have been identified in vertebrates^56^. Interestingly, the Clytia Branch 2 P2XR proteins (ChP2X2–ChP2X4) have all of the amino acids shown to be associated with efficacy of PPADS in blocking P2XR function (Lys70, Asp170, Lys190, and Lys249 in human P2X1^56^); in contrast, all of these amino acids are missing in the Clytia and *Dictyostelium* Branch 1 P2XR proteins with the exception of Lys70 in one of the *Dictyostelium* proteins (Supplementary Fig. S5). Consistently, none of the *Dictyostelium* P2XR channels are inhibited by PPADS^61,62^. Hence, PPADS-sensitivity may allow separation of signaling through Branch 1 and Branch 2 P2XR family proteins in Clytia and other non-bilaterian metazoans.

## Discussion

Extracellular ATP (eATP) released from damaged cells promotes wound healing in vertebrate epithelia^28–31,68^. In vertebrate epithelial cells in vitro, eATP diffusion from the wound site to marginal and submarginal cells induces a wave of intracellular Ca^+2^ beginning in the marginal cells and spreading across the epithelial cell sheet^30,33–37,69^. However, the cellular responses that connect eATP to the healing process are poorly understood. Progress in this field is hindered by: (1) the difficulty of visualizing cellular responses in live vertebrate animals; (2) the problem of teasing out eATP signaling from the complex, overlapping signaling processes taking place in wounded tissues in vivo (i.e. inflammation, cytokine signaling etc.); (3) the potential to miss important aspects of in vivo signaling when using tissue culture models; and (4) the lack of recognized eATP receptors in the most common model systems (*Drosophila, C. elegans*)^59^. Wound healing studies in Clytia overcome these barriers. Clytia is a non-bilaterian metazoan model for epithelial wound healing with many key advantages including its simple anatomy and large epithelial cells that can be imaged in live intact animals, its remarkably fast wound healing, and the conservation of basic wound healing strategies with those seen in more complex animals^21^. Here we use this model to gain better understand eATP signaling in epithelial wound healing, and gain insights into the early evolution of these pathways.

Our finding that inhibition of transcription does not affect the healing process in small wounds in Clytia was initially surprising. Gene expression changes have been described in cnidarians in response to more severe wounds, such as removal of the manubrium in Clytia^70^ and dissection/amputation in *Hydra* and *Nematostella*^18–20^ and wound induced changes in gene expression have been intensively studied in many plant and animal contexts. Nevertheless, in the case of small epithelial wounds in Clytia, where healing is primarily accomplished by lamellipodia-based crawling of marginal cells, strong inhibition of de novo transcription did not affect the healing process. Although we cannot rule out the possibility that residual transcription of key proteins was sufficient for healing, our finding suggests that wound-induced cell polarization, lamellipodia formation, tissue contraction and re-establishment of cell junctions can all be accomplished using existing cellular components. This result is consistent with the extreme rapidity of many of these responses–lamellipodia formation in all wounds in Clytia occur within minutes^21,22^. It is also in agreement with the finding of Lin *et* al. that in the jellyfish *Polyorchis penicillatus* small wounds healed without protein synthesis^71^, and the finding that neither actinomyosin D nor the protein synthesis inhibitor cycloheximide affected lamellipodia formation for at least 3 h in MDCK cells in culture^72^. Amiel et al. argued that inhibition of de novo transcription blocked the earliest stages of wound healing after head removal in *Nematostella*^18^. However, those authors did not assess re-epithelialization at the cellular level. Furthermore, amputation involves damage to multiple tissues and loss of organs, in contrast to the epithelium-specific wound described here. These differences may explain the discrepancies in our findings.

eATP acts as a transcription-independent signal of cell damage throughout the plant and animal kingdoms^59^, and here we show that it promotes epithelial wound healing in Clytia. Our data show that eATP activates a cation-permeable channel in epithelial cells. This suggests that epithelial cells likely respond to wounding in Clytia through eATP-mediated activation of P2XRs. P2XRs are one of only two ATP receptors recognized in animals, and their activation has been shown to allow entry of Ca^+2^ and other cations into cells in a wide variety of animals and tissues^30^. If a P2XR does transduce the eATP signal in wounded Clytia, this becomes the earliest example of eATP involvement in healing via a P2XR-mediated pathway. However*,* despite the ubiquitous use of eATP as a damage signal, not all organisms transduce this signal via P2XR or P2Y receptors. eATP promotes wound healing in photosynthetic plants but they lack P2XR and P2YR proteins^46^. *Dictyostelium* responds to eATP, but its responses are independent of its P2XRs^61–63^; *Dictyostelium* P2XRs appear to regulate osmotic balance through ATP-binding at the vacuole^62^. Our data strongly suggest that wound-released eATP acts through activation of P2XR channels in Clytia exumbrella epithelial cells. However, it remains possible that there are other, undiscovered eATP receptors that permit cation entry into epithelial cells, and mutagenesis of the Clytia P2XRs will be required to prove their essential role.

The cellular targets of eATP signaling in wound healing are poorly understood. Our data show that eATP promotes actin accumulation at the edges of submarginal cells in Clytia, as there was reduced phalloidin staining in submarginal cells when apyrase treatment preceded wounding. Such effects have been previously noted; eATP signaling targeting actin dynamics in marginal cells in the cornea epithelium^37^ and at a distance from a wound in neuroepithelium of frog embryo^38^. Submarginal cells in epithelial wounds are known to form “cryptic lamellipodia” that slide underneath the cell in front of them and are critical for collective cell migration^48,50,51,73^. It is possible that the accumulation of actin we see in Clytia submarginal cells is associated with cryptic lamellipodia formation. One model consistent with our observations would be that eATP released from damaged cells diffuses to the submarginal cells, binds to P2XRs, and promotes actin remodeling via intracellular Ca^+2^ increases, potentially leading to cryptic lamellipodia formation. This model is also consistent with findings that simple diffusion of eATP best accounts for the dynamics of Ca^+2^ increases observed in tissue culture^69^. However, Farooqui and Fenteany^50^ found that apyrase treatment had no impact on the formation of cryptic lamellipodia in MDCK cell monolayers. Further experiments will be needed to define the mechanism of eATP-dependent cellular responses to epithelial wounding in Clytia.

The effect of eATP on the rate of wound closure in Clytia was small but significant and was primarily seen as an increase in the first 5–10 min (Fig. 3). This may be due to a specific eATP effect at the early stages of healing or may reflect rapid degradation of the added ATP by ectonucleotidases^31^. It is also notable that elimination of endogenous eATP through addition of apyrase before wounding slowed but did not block wound healing (Fig. 3). This may be due to incomplete elimination of endogenous eATP. Alternatively, it may indicate that eATP release from damaged cells promotes but is not essential for epithelial wound healing, at least in the small wounds examined in this study. It is possible that the major role of eATP is to send a damage signal to submarginal cells, and these cells play only a minor role in the healing of small wounds. It will be important in future studies to investigate the effects of apyrase on larger wounds where collective cell migration is necessary to close the wound gap.

Phylogenetic analyses identified four P2XR homologs in Clytia. While all four were correctly annotated as P2XRs in the transcriptome, BLAST searches of the genome with vertebrate sequences identified only three of the genes (ChP2X2-4), while searches with the *Dictyostelium* sequences revealed the fourth family member (ChP2X1). Due to low conservation with the vertebrate P2XR proteins, there has been discussion in the literature as to whether *Dictyostelium* proteins are functional P2XR channels. Several reports have now shown that ATP-specific currents are evoked in four of the five *Dictyostelium* proteins when expressed in heterologous systems^61–63^. Thus, Clytia likely contains representatives of two highly divergent types of functional P2XRs.

Identification of two distinct types of P2XR genes in Clytia lead us to re-analyze P2XR evolution using both human and *Dictyostelium* as reference sequences. This approach uncovered two major, deeply divergent branches in P2XR evolution. Branch 1, which includes Clytia ChP2X1, is defined by the *Dictyostelium* sequences, and contains P2XR genes from other single-celled organisms and non-bilaterian metazoans. A second, more recent branch (Branch 2) includes proteins from non-bilaterian metazoans and representatives of animal phyla that span the tree of life. Within Branch 2 there is evidence of multiple expansions, including three Branch 2 P2XR genes forming tandem duplicates in Clytia (ChP2X2-4) and seven P2XR genes in human (P2X1-7). The presence of both Branch 1 and Branch 2 proteins in many cnidarians and in sponges is a novel feature of non-bilaterian metazoans. There are exceptions, however; both Branch 1 and Branch 2 P2XR proteins are encoded in hydrozoan (such as Clytia, *Hydra* and *Nematostella*), scyphozoan, and hexacorallian genomes, but only Branch 2 was found in a cubozoan (*Morbakka virulenta*) and no P2XR sequences were found in a myxozoan (*Thelohanellus kitauei*)). This shows that P2XR is a deeply conserved gene family that exhibits a complex evolutionary history, including many independent expansions and losses in deep eukaryotic and more recent cnidarian evolution.

PPADS inhibition of P2XRs has been a useful tool for probing the mechanism of eATP signaling in many contexts. PPADS sensitivity is species- and gene-specific but is likely limited to the more recent Branch 2 genes. Proteins from *Dictyostelium* and *Ostrococcus* (green alga), both Branch 1 representatives, show ATP-specific currents that are insensitive to PPADS^61–63,74^. In the only functional analysis of a cnidarian P2XR receptor, *Acropora* P2XR showed various physiological responses to eATP when expressed in heterologous systems, all of which were PPADS sensitive^75^ (66). *Acropora* has both Branch 1 and Branch 2 P2XR proteins (Fig. 7), and the protein characterized in this study was the Branch 2 representative. Duan et al.^75^ describe this *Acropora* protein as a P2X7, but it lacks the characteristic C-terminal domain of a P2X7, consistent with the model that P2X7 did not arise until much later in evolution^16^. Interestingly, we found that PPADS injected into the ECM of Clytia prior to wounding had no effect on wound healing (Fig. 3). The insensitivity of wound healing in Clytia to PPADS suggests that ChP2X2–ChP2X4 proteins may not be involved, implicating ChP2X1 as the Clytia P2XR involved in eATP-promotion of epithelial wound healing by default. However further experiments will be needed to test this hypothesis.

Clytia was previously shown to be ideal for imaging live epithelial cells in intact animals during wound healing. In this study we used the cnidarian Clytia to investigate transcriptionally-independent mechanisms that govern epithelial wound healing, focusing on signaling through eATP. We present new protocols to perturb signaling in the ECM, at the epithelial cell surface and in the cytoplasm. Coupled with genomic tools for gene identification and modification^76–82^, and the demonstrated parallels to wound healing in other animals^21^, Clytia is becoming a unique and powerful system for dissecting epithelial wound healing in vivo.

## Methods

### Clytia husbandry

Colonies were grown and maintained using methods described previously^21,22,83^. University of Chicago Clytia colonies were established from Clytia polyps originally gifted from Tsuyoshi Momose and Evelyn Houliston, Observatoire Oceanologique de Villefranche sur Mer, and from Centre National de Resources Biologiques Marines, EMBRC-France (http://www.embrc-france.fr). Baby medusae were collected from these colonies as needed. 3 to 4-week-old Z-11 female medusae were used for all experiments. *Clytia* were maintained in artificial sea water (ASW) (Instant Ocean, Spectrum Brands Blacksburg, VA; 4% Instant Ocean sea salts with specific gravity adjusted to 1.027) at 18 °C in a Z-Hab mini system (Pentair, Minneapolis, MN) with 2-L zebrafish tanks for polyp colonies (Pentair, Minneapolis, MN) and custom-made 5-L kreisel tanks for medusae^22^.

### Exumbrella epithelial wounding

Animals were placed on a depression slide in ASW with the exumbrella epithelium facing the microscope lens. Wounds were made by gently abrading the cell surface with a pipette tip. In some cases, placement of the cover slip was sufficient to generate small wounds. See Lee et al. for details^22^.

### Microinjection

Microinjection needles were made with a Sutter p-97 micropipette puller (Sacramento, CA) using capillary tubes (WPI, Sarasota, FL, TW1004), and broken to produce a tip between 10 and 20 µm. These needles were then backfilled with the indicated volume and concentration of dye or drug. Medusa were placed with the lower epithelium facing up. Using a micromanipulator, the tip of the needle was inserted into the ECM. Pneumatic PicoPump from WPI (Sarasota, FL) was used to inject the dye or drug with an ejection pressure < 10 psi, and no constant hold pressure. For more details see Lee et al.^22^.

### Pharmacology

For all pharmacology experiments, animals were injected with the stated concertation of each drug and allowed to recover in a beaker of ASW for the indicated time before wounding. In all cases Fast Green FCF dye was added before injection (1/50 dilution of 1 mg/ml stock into each reagent).

#### Dyes

3 kDa, 10 kDa, and 70 kDa Fluorescent dextrans (Thermofisher, Waltham, MA) were dissolved in DMSO to make 10 mM, 5 mM, and 250 µM stock solutions, respectively. All stocks were diluted to 100 µM in ASW for injection. 5(6)-CFDA, SE; CFSE (5-(and-6)-Carboxyfluorescein Diacetate, Succinimidyl Ester (CFDA), (Thermofisher, Waltham, MA) was made as a 100 mM stock solution in DMSO and diluted to 100 µM in ASW for injection or bath application. FM 4–64 (Thermofisher, Waltham, MA), was dissolved in DMSO to make a 10 mM stock solution and diluted to 10 µM in ASW for injection or bath application. YO-PRO-1 was dissolved in DMSO to create a 1 mM stock solution and diluted to 150uM in ASW for injection.

#### ATP agonists and antagonists

Both ATP disodium salt (Sigma, St. Louis MO) and ADP sodium salt (Sigma, St. Louis MO) were dissolved in purified MQ water to make 100 mM stock solutions. ATPγS tetralithium (ATPγS, Tocris, Bristol UK) was dissolved in MQ water to make a 50 mM stock. ATP, ATPγS and ADP stocks were diluted to 1 mM (ATP, ATPγS) or 5 mM (ADP) in ASW for injection^52^. For the pH effects experiment, 1 mM ATP was adjusted to pH 8.2 with NaOH. BzATP triethylammonium (Tocris, Bristol UK) was dissolved in MQ water to make a 10 mM stock and diluted to 300 uM in ASW for injection^84^. Oxidized ATP (oATP) (EMD Millipore, Burlington, MA) was dissolved in MQ water to make a 1 mM stock and then diluted to 100uM in ASW for injection^85^. PPADS sodium salt (Cayman Chemical, Ann Arbor, MI) was dissolved in ASW to create a 50 mM stock and diluted to 1 mM for injection^86^.

#### Drugs

Actinomycin D (Cayman Chemical, Ann Arbor, MI, 11,421) was dissolved in DMSO (Thermo Fisher, Carlsbad, CA, D12345; 10 mg/mL) then diluted with ASW. 80 mM ActD was microinjected into the ECM of each of the four quadrants, defined by the radial canals, of adult medusa. Diluted DMSO was injected as a control. Animals were incubated in ASW for 2-h post-injection. Apyrase (NEB, Ipswich, MA) supplied as a 500U/ml stock solution was diluted to 10U/ml in ASW for injection^87^.

### Microscopy and timelapse movies

For dextran experiments, whole animals were imaged on a Zeiss, Stemi SV 11 Apo Stereoscope (Oberkochen, Germany). Wound healing was imaged using a Leica DMR microscope (Wetzlar, Germany) with standard DIC optics. Images were captured using Zen on an AxioCam 506 mono (ZEISS). Fluorescence images were obtained on the same microscope with a halogen lamp as the light source and standard Leica filter cubes for DAPI, FITC, and Rhodamine. For time lapse movies, pictures were taken at 11 second intervals and processed to create movies using FIJI (http://imagej.net/Fiji/Downloads). During processing, movies were registered using the FIJI plugin Linear Stack Alignment with SIFT. See Lee et al. for details^22^.

### Rate of wound closure

To measure the rate of wound closure, the wound area was measured every 10 frames using FIJI (http://imagej.net/Fiji/Downloads) and plotted using Prism 9 (GraphPad, La Jolla, CA).

### YO-PRO-1 uptake

15 µM YO-PRO-1 was injected in one quadrant and allowed to diffuse. After 20 min, 1 mM ATP or ASW control was injected into two quadrants. Some animals were left as YO-PRO-1 only controls. Before imaging, medusae were incubated in 20 µM Hoechst. For quantitation of YO-PRO-1 uptake, each quadrant of the medusa bell was imaged in two areas in the green channel (YO-PRO-1) and the blue channel (nuclei, Hoechst 33342). FIJI plugin ComDet 5.5 was used to identify YO-PRO-1 fluorescent particles in the cytoplasm and Hoechst-stained nuclei. We specified a particle size constraint and an intensity threshold for all images to determine the number of YO-PRO-1 particles and the number of nuclei in an image. The number of YO-PRO-1 particles was normalized by the number of nuclei per image. These values were then averaged in each jellyfish.

### De novo transcription

To confirm the effectiveness of Actinomycin D treatment, de novo transcription was detected by incorporation of EU into RNA using Click-iT® RNA Imaging Kit (Thermo Fisher, Eugene, OR, Click-iT® RNA Imaging Kit C10330). Animals were incubated in EU (1 mM) for 2 h, then fixed with 3.7% formaldehyde diluted from 37% stock with ASW. Post-fixation, animals were washed 3 times in ASW then processed according to the kit protocol with the addition of extra wash steps (Click-iT® Reaction Rinse Buffer 2 × 5 min; ASW 2 × 5 min). Finally, DNA was stained with 20 µM Hoechst 33342 before mounting in glycerol. To quantify de novo transcription, masks were made from images of Hoechst staining to localize the nuclear regions. Fluorescence intensity from incorporated EU was then quantified in these regions to measure transcription.

### Actin accumulation

For actin visualization, medusae were injected with ASW or 10U Apyrase and incubated for 30 min. Then animals were wounded as described above. Ten minutes post-wounding, medusae were fixed in 3.7% formaldehyde in ASW, washed in ASW, and permeabilized with 0.1% Triton X-100. The medusae were then stained overnight in Alexa FluorTM Phalloidin 488 (Invitrogen) (300U/1.5 ml in methanol diluted 1:200 in ASW). The fixed medusae were mounted on a depression slide in 80% glycerol, 20% ASW, and 20 μM Hoechst dye solution.

For actin analysis, wounds were imaged. For each image, 4–8 lines were drawn using FIJI (http://imagej.net/Fiji/Downloads) perpendicular from the wound edge into the field of cells. Areas with obvious folded tissue or regions out of focus were avoided. For each line, the intensity over the length (plot profile) was measured and stored as CSV files. This data was processed by in-house code to adjust the baseline of the intensity values and find local maxima using a peak width between 20 and 40 µm. Maxima corresponded to cell edges. The ratio of the maxima of 3 sub-marginal cells to the maxima of the cell 1 at the wound margin were calculated. Scripts for actin wound analysis are provided as a Python Juypter notebook.

### Statistical analysis

Statistical details of experiments can be found in figure legends. n represents the number of medusae, cells, or wounds as specified in the legends. Data are shown as means ± SEM. All statistical analysis was performed in Prism 9.

### Phylogenetic analysis

Protein gene model FASTAs and genome gffs were downloaded from public databases or publication-related data repositories (Supplementary Tables S1 and S2). FASTA sequences were filtered to retain only the longest isoform per gene based on header or gff information. Genome completeness was evaluated using BUSCO (5.3.0)^88^ and its Metazoa database. Target genomes were selected based on genome quality and phylogenetic representation relative to Clytia and across animals, and also on previous studies in specific species. Final 17 species included were Viridiplantae *Ostreococcus tari*, Amoebozoa *Dictyostelium discoideum*, Fungi *Allomyces macrogynus*, Choanoflagellata *Monosiga brevicollis*, Porifera *Amphimedon queenslandica*, Ctenophora *Mnemiopsis leidyi*, Placozoa *Trichoplax adhaerens*, Cnidaria Myxozoa *Thelohanellus kitauei*, Cnidaria Cubozoa *Morbakka virulenta*, Cnidaria Hexacorallia *Nematostella vectensis*, Cnidaria Hexacorallia *Acropora millepora*, Cnidaria Scyphozoa *Rhopilema esculentum*, Cnidaria Hydrozoa *Clytia hemisphaerica*, Mollusca *Lottia gigantea*, Arthropoda *Limulus polyphemus*, Cephalochordata *Branchiostoma floridae*, Chordata *Homo sapiens*. A reference gene set FASTA was built using human HUGO Gene Nomenclature Committee-identified P2XR sequences (HUGO:https://www.genenames.org/) and published sequences for *Dictyostelium discoidia*. Local BLASTp databases were built for individual target and reference genomes (Blast + 2.6.0) (Supplementary Data). Reference sequences were BLASTed against each genome using BLASTp (e-value threshold e-1). All initial hits in target genomes were BLASTed against the reference genome. Initial hits having a top hit to a reference gene in a reference genome were retained and formed the candidate gene set. Candidate sequences were combined with reference sequences. The combined sequences were aligned in MAFFT (7.515)^89^ and the alignment trimmed in ClipKit (1.4.1)^90^. Maximum-likelihood-based phylogenetic trees were built using the trimmed alignments and IQTree (2.1.4) tree-building software, including use of ultrafast bootstraps. Alignments and trees were evaluated by hand in Geneious (2023.0.4) (geneious:https://www.geneious.com), FigTree (1.4.4) (https://github.com/rambaut/figtree), and iTOL (6.7.3)^91^. Partial sequences were identified in alignments and removed from the combined set of sequences to produce a final gene set. The final gene set was aligned, trimmed, and trees built as before for final analysis. The final tree was evaluated in iTOL^91^ and branches with less than 95% ultrafast bootstrap support were collapsed. Scripts for all steps post-genome evaluation are provided as a Python Juypter notebook.

### Supplementary Information

Supplementary Information 1.

Supplementary Information 2.

Supplementary Information 3.

Supplementary Information 4.

Supplementary Information 5.

Supplementary Information 6.

Supplementary Information 7.

Supplementary Information 8.

## Data Availability

All data generated or analyzed during this study are included in this published article (and its Supplementary Information files).
