# Supplementary material for: Epithelial wound healing in Clytia hemisphaerica provides insights into extracellular ATP signaling mechanisms and P2XR evolution
Source: Sci Rep. 2023 Nov 1;13:18819. doi: 10.1038/s41598-023-45424-5 (PMC10620158; doi:10.1038/s41598-023-45424-5)
Supplement: Supplementary file 8 — Supplementary Information 8. [file 41598_2023_45424_MOESM8_ESM.pdf]

Supplementary Figures

Epithelial wound healing in Clytia hemisphaerica provides insights into extracellular ATP signaling mechanisms and P2XR evolution

Elizabeth EL Lee[1], Isabel O'Malley-Krohn[2], Eric Edsinger[3], Stephanie Wu[2], Jocelyn Malamy[1#]

1.Department of Molecular Genetics and Cell Biology, The University of Chicago, 929 East 57th Street, Chicago, IL 60637
2. Biological Sciences Collegiate Division, The University of Chicago, 929 East 57th Street, Chicago, IL 60637
3. Whitney Laboratory for Marine Biosciences, University of Florida, 9505 N Ocean Shore Blvd, St. Augustine, FL 32080

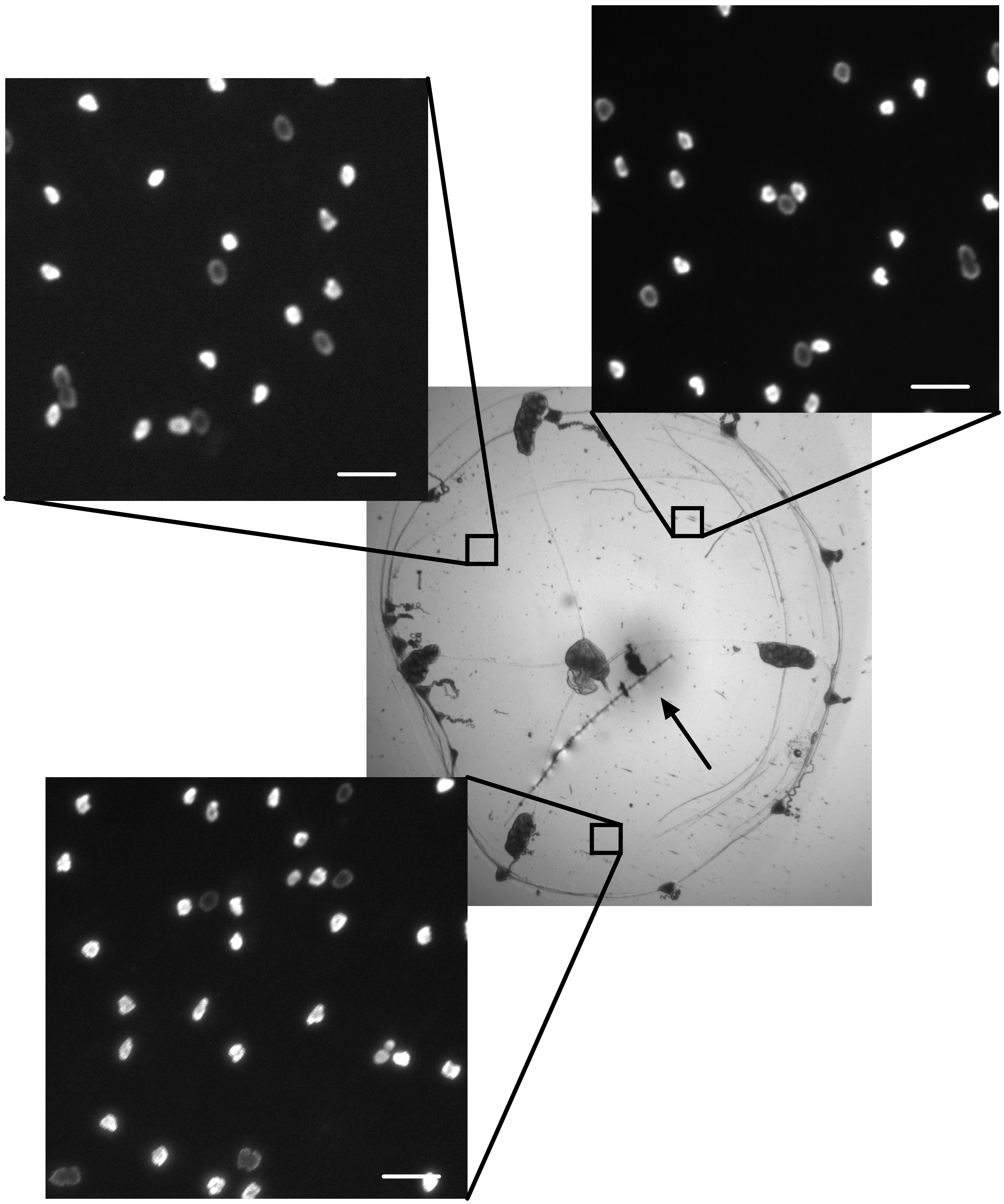

Supplementary Figure S1. Hoechst dye diffuses from the site of injection to all four quadrants

Adult medusa injected with 10 µM Hoechst dye. Injection site is indicated by an arrow. Inserts are images of Hoechst-stained nuclei in three locations 5 minutes post-injection, indicating diffusion of the dye throughout the mesoglea and into the cells. Scale bars = 50 µm.

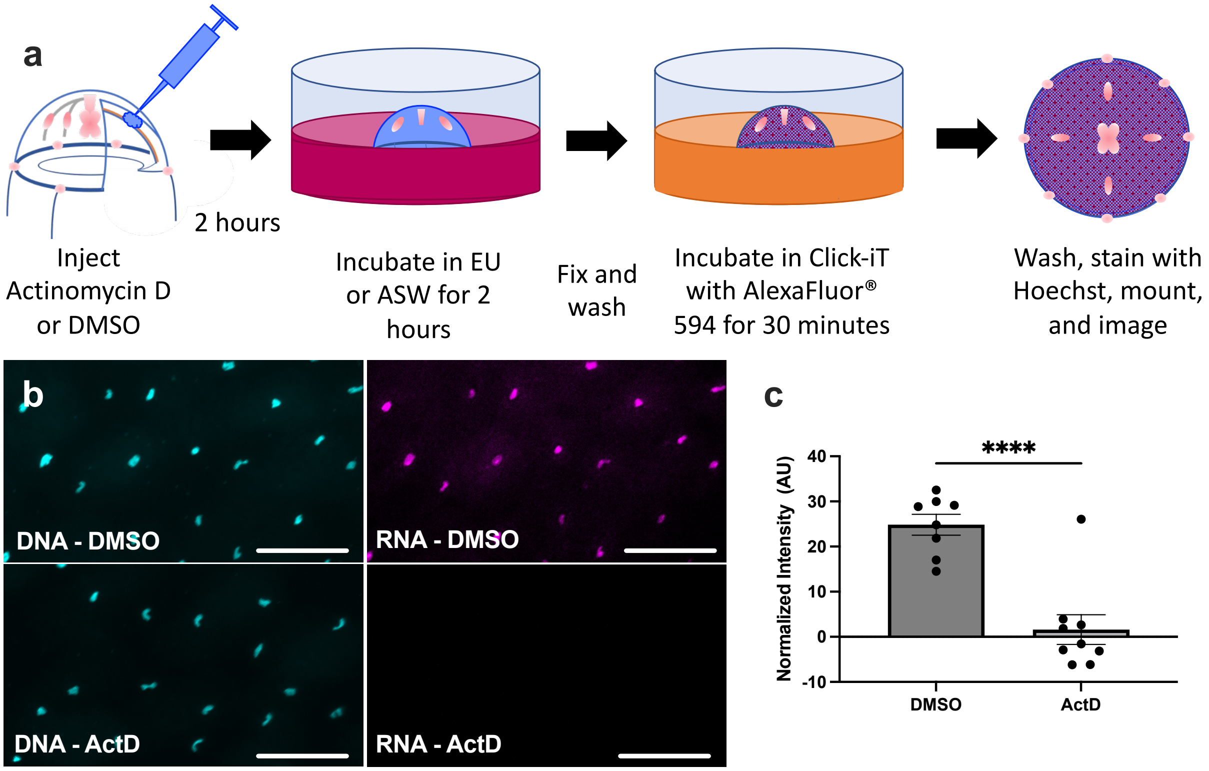

Supplementary Figure S2. Actinomycin D inhibits *de novo* transcription in Clytia.

a) Schematic representation of EU labeling of *de novo* transcription in the presence of actinomycin D (ActD) or DMSO. **b)** 80 mM ActD represses transcription in the exumbrella epithelial cells. Representative images of exumbrella epithelial cells show DNA (Hoechst (left)) or *de novo* transcripts (RNA, EU (right)). **c)** Quantification of *de novo* transcription in the presence or absence of 80 mM ActD. Each datapoint represents the mean fluorescence intensity of EU in Hoechst-labeled nuclei in a single animal; two images were collected per animal. DMSO: n = 8, ActD: n = 9. n = number of animals. Bars represent the mean of the datapoints $\pm$SEM Unpaired two-tailed T-test: P-value <0.0001 (****).

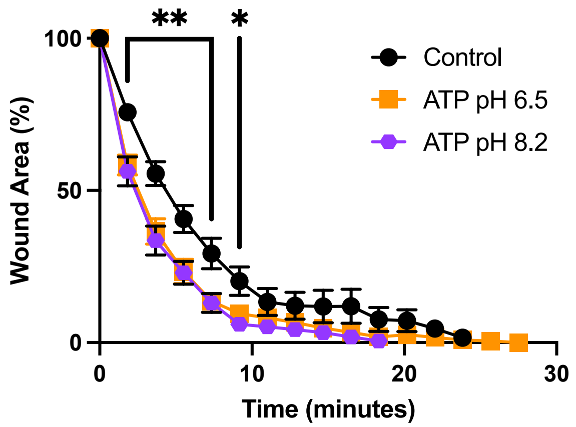

Supplementary Figure S3. Reduced pH is not responsible for ATP's effect on wound healing rate.

ASW (control) has a pH of 8.2, whereas unadjusted 1 mM ATP has a pH of 6.5. Adjusting 1 mM ATP to pH 8.2 does not alter its ability to increase the wound healing rate, indicating that weak acidity of the solution does not explain the observed effect. One-way analysis of variance (ANOVA) at each time point: $P < 0.01$ (**); $P < 0.05$ (*). n = number of wounds analyzed in independent animals. Data is presented as mean percent of original wound area ± SEM.

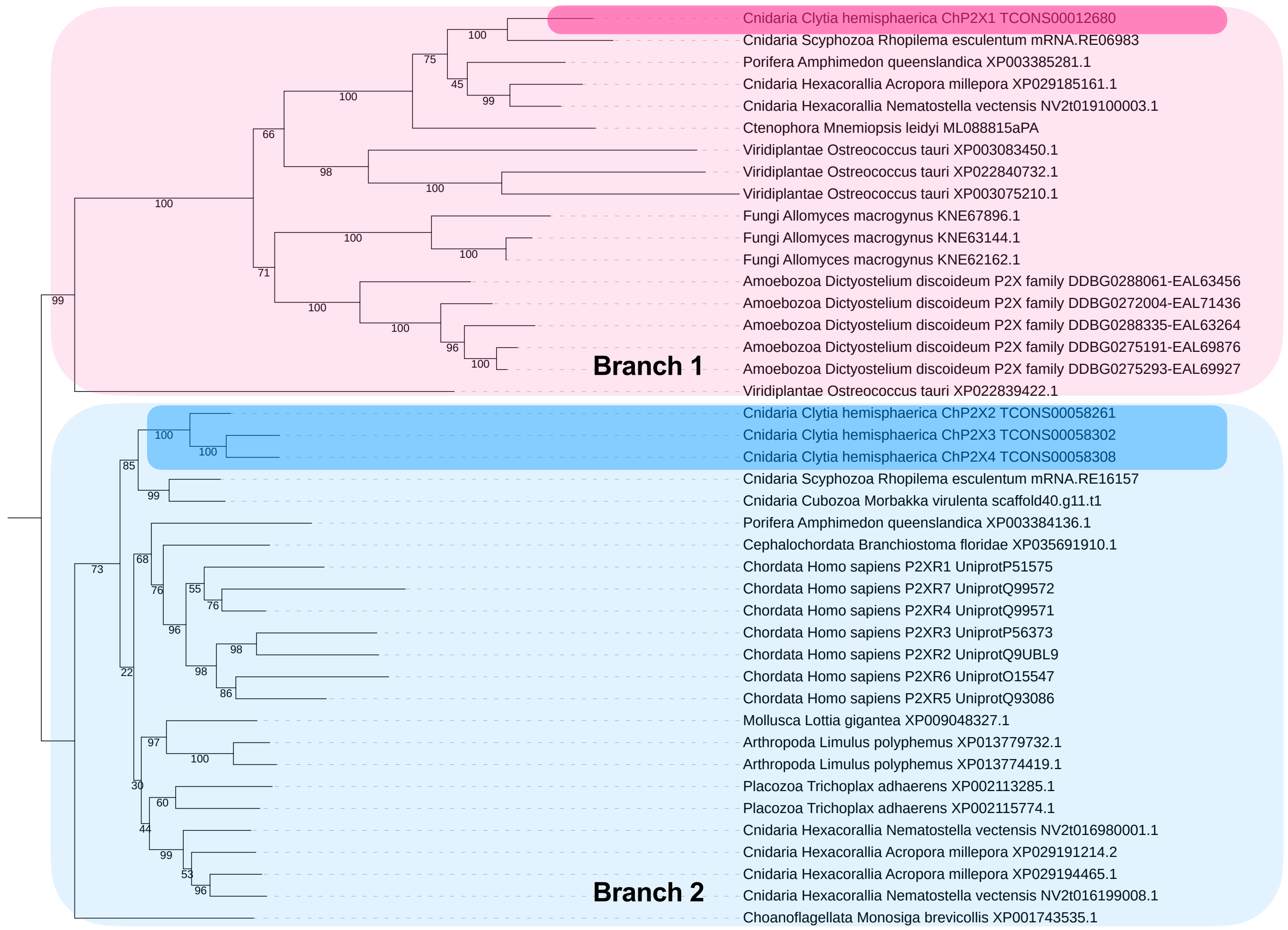

Supplementary Figure S4. Un-rooted un-collapsed phylogenetic tree of P2XRs across eukaryotes. Homologs were detected by reciprocal BLAST back to reference gene family. Sequences were aligned in MAFFT, trimmed in ClipKit, and maximum likelihood trees produced in IQTree (see Methods). The tree is unrooted and no branches are collapsed. Branches having 95% or greater ultrafast bootstrap support are considered significant under ultrafast bootstrap support are considered significant under ultrafast bootstrapping guidelines[91]. A rooted collapsed version of the tree is provided as Figure 7 in the main text.

```
Conservation                               :: * * :   ::::::.  .  : . :: * :    .
Human P2X1         1 ----------MARRFQEELAAFLFEYDTPRMVLVRNKKVGVIFRLIQLVVLVYVIGWVFL 50
Clytia_00058302    1 LKMNQSRKQMVLEKSKFHCINFLLSYETGKVVEIQNKKVAFWYRLIQLSVIIYVIGYAII 60
Clytia_00058261    1 ---------MGLTECRQKGIGILLHYETVKIIDIKNKKVGALYRFIQLIIISYVIGYVIV 51
Clytia_00058308    1 --------MTGCAKVKNFGYDFLFNYQTVKLAEIKDKKVGLSFRVIQLAIVCYIIFYVIV 52
Clytia_00012680    1 --------------------MSLFHYVTYKYIIIKDKRLGIAYYILAFLIIFYTLVQLLV 40
DICTYP2XE          1 ------------MNFRNIDWDSLFSYSTIKIVRIRDKRLGILHFAFLIGIILYIIVGTIF 48
DICTYP2XD          1 -----------------MDWDNIFSYNTAKIVTIKDRRLGGLHIIFMVLIIVYIVIYSTI 43
DICTYP2XA          1 -------------MGFSFDWDDIFQYSTVKIVRIRDRRLGILHLSFLVGIVAYIVVYSAI 47
DICTYP2XB          1 ---------------MTIDWDSILSYNTIKVVRIRDRRLGILHLCFLIVIVLYVVVYSAI 45
Conservation          ..     .   ..    :
Human P2X1        51 YEKGYQTSSGLISSVSVKLKGLAVTQLPG---------------------L------GPQ 83
Clytia_00058302   61 YDKGYQEIDSAISSTNTKVKGITHVNFGN---------------------FPSTFFNGRM 99
Clytia_00058261   52 YKKGYQEFDVAISTVTTKLKGTTYVDFGK---------------------FNSTLFNGIR 90
Clytia_00058308   53 YKKGYQEFDTAISTISTKMKGITHVDFTG---------------------FNSSLFNGLQ 91
Clytia_00012680   41 NKSYLQYDSSPSGNMRILVNTPTKLQNDENEYNINPDYCKQKESA-----NST----ICR 91
DICTYP2XE         49 LQKKYLVLESPIGSIRTSLMAPSVKPT-------DLPYCLKNGTDTSYDGYPNK---PCQ 98
DICTYP2XD         44 YKKGYLLTETPVGSIRASLLAPNEFKDDS-----NFKYCDDN---LIEYNFTKL---ECD 92
DICTYP2XA         48 IKKGYLFTEVPIGSVRTSLKGPNTFAS-------NLTYCSNQQHNGSTYPFTPL---ECN 97
DICTYP2XB         46 IKKGYVTTEEPVGSIRTSLLAPDELKS-------NQAYCKNN---TEPYPYEKL---DCV 92
Conservation           *                         .    *      ..
Human P2X1        84 VWDVADYVFPAQGDNSFVVMTNFIVTPKQTQGYCAE----HPEG--GICKEDSGCTPGKA 137
Clytia_00058302  100 IYDPQDYVIK-QDGDSFFVMTNMVITPKQYQGKCPE----DPESYRTICKTDKDCQQGTS 154
Clytia_00058261   91 IYDPSDYVIPPQETDAVFVMTNMILTPKQSQSNCPE----DSKFKDIHCKKDSDCPKMVP 146
Clytia_00058308   92 VYDAEDYVIKPKDGNSLFVMTNMVITPKQYQGKCPE----DPESYQALCKTDKDCQQETS 147
Clytia_00012680   92 KLDGLSINWPIESRSVTLAT-FIKEK-WQRPKDCQYALVYDPEN---PVKYE-------- 138
DICTYP2XE         99 YWDEYLVLYPPSEESSMFITTRCTQETQSTVNGCNL---SEP-----TCVYN-------- 142
DICTYP2XD         93 YYDEAFVSFPVGDDVSFAVTTRVKTL--DQVLNCSS---KNP-----KCKYT-------- 134
DICTYP2XA         98 YWDEQLALFPVGQDSTFTCTTRVRLS--KQEANCNF---TDP-----TCKFV-------- 139
DICTYP2XB         93 YYDEKLALFPIGDDVGFTASTRMRIS--DQTVNCSL---MNP-----SCKFY-------- 134
Conservation                                                                :
Human P2X1       138 KRKAQGIRTGKCVAF--NDTVKTCEIFGWCPVEVDDDIPRP---------ALLREAENFT 186
Clytia_00058302  155 NKYGHGERSGRCVPSDRNSTVKVCESYGWCPTEYDV-LPMENYNNFSKTIPLLDGTKEST 213
Clytia_00058261  147 VKNGNGVRTGKCVSSDRNSSMTVCEIYGWCPTEVDQ-LPMPN-NNFSDTVPLLDGTKYST 204
Clytia_00058308  148 NKYGHGERTGRCVPSYRNSTLTVCEIHGWCPTEDDR-LPMIK-SNVSTNIPLLEGTKHAT 205
Clytia_00012680  139 ----DL----------------------------------------RDVDYYAVNPEFAL 154
DICTYP2XE        143 ----TT----------------------------------------SSSDFYIANVENFT 158
DICTYP2XD        135 ----TV----------------------------------------STRNVYVSDIEDFT 150
DICTYP2XA        140 ----DE--------------------------------PG------SAKNIYIADIESFT 157
DICTYP2XB        135 ----TN----------------------------------------TSMNVYLADIESFT 150
Conservation         : :.: :                 .   :     .   .                   :
Human P2X1       187 LFIKNSISFPRFKVNRRNLVEE-VNAAHMKTCLFHKTLHP--------------LCPVFQ 231
Clytia_00058302  214 VLIKNQIHFPKFKVTRNNIVHNQKKGSYMKRCLHNEETDP--------------FCPIFK 259
Clytia_00058261  205 MLVKNQISFPKFRAIRNNIIQF-NDSTYLKRCHYNSDTDP--------------LCPIFN 249
Clytia_00058308  206 VLIKNQIDFMKFDVATYNIPNT-NNRTYMKKCRYNAKTDP--------------LCPIFR 250
Clytia_00012680  155 IKVEHSFTAFSGQLT--------HSHRRMSGSLVDMNGNTIKRL---NSNGVSKVDK-IL 202
DICTYP2XE        159 ILLDHTLSAPSLGIQ--------YNGAQLNGQLLDTDGNPMSLPP-PNIVGVKGSPDIMS 209
DICTYP2XD        151 ILIDHTMFAPSSLIQ--------YNSKQLKGYILDNDNNEIQINETINTVGIPGKPDILT 202
DICTYP2XA        158 ILIDHTMYASSSGSQ--------FNAVDLHGYILNQDGDEVQIDANGTSIGVSGKPDIMT 209
DICTYP2XB        151 VLIDHTMYAPSSQIQ--------FNGDDLSGYVLDQNGNEIQLNESVNTIGVQGKPDILQ 202
Conservation         :  .:                 .   .  .* :    * :                * ..
Human P2X1       232 LGYVVQES------------GQNFSTLAEKGGVVGITIDWHCD--LDWHVRHCRPIYEFH 277
Clytia_00058302  260 LRNIVENC------------GDNYTKVGFKGGIYGIIIKWDCN--LDHSIYDCVPEYKFE 305
Clytia_00058261  250 LEDIVSSC------------GDNYTDVAYQGAIYGIIIEWDCN--LDHSIDECLPKYQFK 295
Clytia_00058308  251 LQDIVSSC------------GDNYTQVAFKGAIYGIIIEWKCN--LDYSIEECLPKYRFK 296
Clytia_00012680  203 LKDFLIAANV-SLDEVSDALNAKERTFRQNGLILHVSIMYKNSEKMLYGVNIPEYTYHVR 261
DICTYP2XE        210 LQGVLTAAGVESLDSQ--GLANKSRTIRDDGILILCFITYSNT--YTYNTGNYHYTYQFK 265
DICTYP2XD        203 IGKLLQLANI-DLDGA--SSVNSTNSVRYDGVVALVFITYSNT--FSYNTNNFKYVYSIQ 257
DICTYP2XA        210 IGQLLSFGGV-SLDQA--SPVDSNVSIRYDGVVLFVFITYSNT--YTYSTSDFKYVYSVQ 264
DICTYP2XB        203 LGKLLEFAGV-DLDGP--SLVNSSNSIRYDGCVLFVFIEYSNT--FSYDLKKIKYVYSIK 257
Conservation          :            :             .   *   . ..::. :   *  *.*     :
Human P2X1       278 GLYEE-KNLSPGFNFRFARHFV----ENGTNYRHLFKVFGIRFDILVDGKAGKFDIIPTM 332
Clytia_00058302  306 RLDDEKTPLSHGYNFRYVNYFV----ENGTRYRTLTKAYGIKFELVVHGEAGKFSFIPLM 361
Clytia_00058261  296 RLDDVNSPISPGYNFRYADYYV----EEDTRYRVLTKAYGIKFELLVHGKAGKFNVIPLL 351
Clytia_00058308  297 RLDNSRSSISKGFNFRYVNYYV----EKGKRYRTLTKAFGIKFELLAHGKAGKFNIIPLL 352
Clytia_00012680  262 RVPFSD--------YRLKQEIPIANNEGNICERIIKKRYAVRIEFHQGGKIGQFSSANLV 313
DICTYP2XE        266 LVQNTK--------YKIVEPVF----TKDVEDRYIFDRHGVRIIFIQTGQLGQFDFQTML 313
DICTYP2XD        258 KVEDTE--------YGVPEAVI----LDNVSSRMYYNRHGIRLIFIQNGEIGSFNFQALL 305
DICTYP2XA        265 QIANTI--------YDVPETII----LESIHSRLLYKRHGIRVIFIQTGTIGSFHFQTLL 312
DICTYP2XB        258 KVDDTA--------YDVPEVII----LNNENSRLYYKRHAIRLIFIQTGVIGSFNFQSLL 305
Conservation           : : :.:.  :: : * :   :  ::  * .
Human P2X1       333 TTIGSGIGIFGVATVLCDLLLLHILPKRHYYKQKKFKYAEDMGP-----GAAERDLAATS 387
Clytia_00058302  362 LNLGAGIGLLGIATFWCDFIVLNLAKKRNVYRNHICESVGRGMSSNEKSSLDGDTK---- 417
Clytia_00058261  352 TNLGAGMALLGIATVWCDFIVMYLLKKRNVYREHKYQRVDDDSQDPKGNVQVDDDENE-- 409
Clytia_00058308  353 TNLGAGIGLLGLATLCCDFIVLNLVPKRNVYRDYICADVTQEYEEHLREAAKEEKEEATR 412
Clytia_00012680  314 FQLVSVMGLLTLTATVIDVIALYIVPNKDNYRQYVFDESPEFSEQEKQTLKENVNDEQ-S 372
DICTYP2XE        314 LTFVSGIGLVTAASLIVDIIATRIMPQRSRYQELKFQDSSINN----TQKTPTNDHTP-L 368
DICTYP2XD        306 LTFVSGLGLLAISTVLVDQLAIRLFPERKTYSSHKFQITHGFSESRNKLRISQNEKDP-L 364
DICTYP2XA        313 LTLVSGLGLLAVATTVVDQLAIRLLPQRKSYSSLKFQVTESMSNPMKKRITTDEGEDV-L 371
DICTYP2XB        306 LTLVSGLGLLTVSTLIVDQLAIRFLPQRKSYSSLKFQTTESF--RMKKKIVNDDGEDK-L 362
Conservation
Human P2X1       388 ST---LGLQENMR--TS-------------------------- 399
Clytia_00058302  418 ---------QNCD--EKKTQEEN-------------------- 429
Clytia_00058261  410 LE-------------SDRPEAER-LIKP--------------- 423
Clytia_00058308  413 LK---LMMVNKCA--VRSPHTARKLLEML-------------- 436
Clytia_00012680  373 KKDQ--------------------------------------- 376
DICTYP2XE        369 LKDNEDTINENSYQNNSYEK----------------------- 388
DICTYP2XD        365 LLVETTKNNENNNNNDDYNDDDNEIFDDNNNGYQNIQNNNIIL 407
DICTYP2XA        372 YT-RIEGL----------------------------------- 378
DICTYP2XB        363 YH-NIEAL----------------------------------- 369
```

Supplementary Figure S5. Alignment of Clytia and Dictyostelium P2XR homologs with human P2XR.

Sequences were aligned using Clustal omega (https://www.ebi.ac.uk/Tools/msa/clustalo/)[S1]. Red boxes indicate residues similar or identical across the tree of life as identified by Surprenant and North[66]. Similar are IVLM, FYWH, KRH, EQDN. Yellow highlighting indicates amino acids shown to be essential for ATP binding in vertebrates. Blue boxes identify critical amino acids for PPADS sensitivity in vertebrates. Note that ChP2X1 does not have the critical amino acids for PPADS sensitivity.

Supplementary Table S1 – Metazoan species genomes evaluated for completeness.

Original list of species whose genomes were evaluated for completeness.

Genus species	Name	Phylo-name
Acanthopleura granulata	Chiton	Metazoa-Mollusca-Polyplacophora-Chitonida-Chitonidae-Acanthopleura-granulata
Achatina fulica	Giant African snail	Metazoa-Mollusca-Class_unresolved-Order_unresolved-Family_unresolved-Achatina-fulica
Acropora millepora	Stony coral	Metazoa-Cnidaria-Anthozoa-Scleractinia-Acroporidae-Acropora-millepora
Adineta ricciae	Rotifer	Metazoa-Rotifera-Eurotatoria-Adinetida-Adinetidae-Adineta-ricciae
Ailuropoda melanoleuca	Panda	Metazoa-Chordata-Mammalia-Carnivora-Ursidae-Ailuropoda-melanoleuca
Alligator mississippiensis	Alligator	Metazoa-Chordata-Class_unclassified-Crocodylia-Alligatoridae-Alligator-mississippiensis
Amblyraja radiata	Thorny skate	Metazoa-Chordata-Chondrichthyes-Rajiformes-Rajidae-Amblyraja-radiata
Amia calva	Bowfin fish	Metazoa-Chordata-Actinopteri-Amiiformes-Amiidae-Amia-calva
Amphibalanus amphitrite	Barnacle	Metazoa-Arthropoda-Hexanauplia-Sessilia-Balanidae-Amphibalanus-amphitrite
Amphimedon queenslandica	Marine sponge	Metazoa-Porifera-Demospongiae-Haplosclerida-Niphatidae-Amphimedon-queenslandica
Amyelois transitella	Navel orangeworm moth	Metazoa-Arthropoda-Insecta-Lepidoptera-Pyralidae-Amyelois-transitella
Amynthas corticis	Earthworm	Metazoa-Annelida-Clitellata-Crassiclitellata-Megascolecidae-Amynthas-corticis
Anneissia japonica	Crionoid	Metazoa-Echinodermata-Crinoidea-Comatulida-Comatulidae-Anneissia-japonica
Anopheles gambiae	Mosquito	Metazoa-Arthropoda-Insecta-Diptera-Culicidae-Anopheles-gambiae
Aphelenchus avenae	Aphelencus nematode Free	Metazoa-Nematoda-Chromadorea-Rhabditida-Aphelenchidae-Aphelenchus-avenae
Apis mellifera	Western honey bee	Metazoa-Arthropoda-Insecta-Hymenoptera-Apidae-Apis-mellifera
Apolygus lucorum	True bug	Metazoa-Arthropoda-Insecta-Hemiptera-Miridae-Apolygus-lucorum
Apostichopus japonicus	Japanese spiky sea cucumber	Metazoa-Echinodermata-Holothuroidea-Aspidochirotida-Stichopodidae-Apostichopus-japonicus
Aptenodytes patagonicus	King Penguin	Metazoa-Chordata-Aves-Sphenisciformes-Spheniscidae-Aptenodytes-patagonicus
Archivesica marissinica	Deep sea clam	Metazoa-Mollusca-Bivalvia-Venerida-Vesicomyidae-Archivesica-marissinica
Argiope bruennichi	Wasp spider	Metazoa-Arthropoda-Arachnida-Araneae-Araneidae-Argiope-bruennichi
Aricia agestis	Brown argus butterfly	Metazoa-Arthropoda-Insecta-Lepidoptera-Lycaenidae-Aricia-agestis
Artemia franciscana	Brine shrimp	Metazoa-Arthropoda-Branchiopoda-Anostraca-Artemiidae-Artemia-franciscana
Artibeus jamaicensis	Jamaican fruit bat	Metazoa-Chordata-Mammalia-Chiroptera-Phyllostomidae-Artibeus-jamaicensis
Arvicola amphibius	European water vole	Metazoa-Chordata-Mammalia-Rodentia-Cricetidae-Arvicola-amphibius
Asterias rubens	Common starfish	Metazoa-Echinodermata-Asteroidea-Forcipulatida-Asteriidae-Asterias-rubens
Astyanax mexicanus	Mexican tetra blind cavefish cave	Metazoa-Chordata-Actinopteri-Characiformes-Characidae-Astyanax-mexicanus
Austrofundulus limnaeus	Thickset riviulus killifish Guppies	Metazoa-Chordata-Actinopteri-Cyprinodontiformes-Rivulidae-Austrofundulus-limnaeus

Bagarius yarrelli	Goonch catfish	Metazoa-Chordata-Actinopteri-Siluriformes-Sisoridae-Bagarius-yarrelli
Balaenoptera acutorostrata_scammoni	Minke whale	Metazoa-Chordata-Mammalia-Artiodactyla-Balaenopteridae-Balaenoptera-acutorostrata_scammoni
Balaenoptera musculus	Blue whale	Metazoa-Chordata-Mammalia-Artiodactyla-Balaenopteridae-Balaenoptera-musculus
Balaenoptera physalus	Fin whale	Metazoa-Chordata-Mammalia-Artiodactyla-Balaenopteridae-Balaenoptera-physalus
Bathymodiolus platifrons	Deep sea mussel	Metazoa-Mollusca-Class_unresolved-Order_unresolved-Family_unresolved-Bathymodiolus-platifrons
Batillaria attramentaria	Japanese mud snail	Metazoa-Mollusca-Gastropoda-Order_unclassified-Batillariidae-Batillaria-attramentaria
Belgica antarctica	Antarctic midge	Metazoa-Arthropoda-Insecta-Diptera-Chironomidae-Belgica-antarctica
Biomphalaria glabrata	Ram's horn snail	Metazoa-Mollusca-Gastropoda-Order_unclassified-Planorbidae-Biomphalaria-glabrata
Blattella germanica	German Cockroach	Metazoa-Arthropoda-Insecta-Blattodea-Ectobiidae-Blattella-germanica
Boleophthalmus pectinirostris	Mudskipper Gobies	Metazoa-Chordata-Actinopteri-Gobiiformes-Gobiidae-Boleophthalmus-pectinirostris
Bombyx mori	Domestic silk moth	Metazoa-Arthropoda-Insecta-Lepidoptera-Bombycidae-Bombyx-mori
Bos taurus	Cow	Metazoa-Chordata-Mammalia-Artiodactyla-Bovidae-Bos-taurus
Branchiostoma floridae	Amphioxus	Metazoa-Chordata-Leptocardii-Amphioxiformes-Branchiostomidae-Branchiostoma-floridae
Brugia malayi	Genetic model nematode III	Metazoa-Nematoda-Chromadorea-Rhabditida-Onchocercidae-Brugia-malayi
Bufo gargarizans	Asiatic toad	Metazoa-Chordata-Amphibia-Anura-Bufonidae-Bufo-gargarizans
Bugula neritina	Brown bryozoan	Metazoa-Bryozoa-Gymnolaemata-Cheilostomatida-Bugulidae-Bugula-neritina
Caenorhabditis elegans	Genetic model nematode V	Metazoa-Nematoda-Chromadorea-Rhabditida-Rhabditidae-Caenorhabditis-elegans
Caligus rogercresseyi	Sea louse copepod	Metazoa-Arthropoda-Hexanauplia-Siphonostomatoida-Caligidae-Caligus-rogercresseyi
Callorhinchus milii	Australian ghost shark	Metazoa-Chordata-Chondrichthyes-Chimaeriformes-Callorhinchidae-Callorhinchus-milii
Camelus dromedarius	Camel	Metazoa-Chordata-Mammalia-Artiodactyla-Camelidae-Camelus-dromedarius
Candidula unifasciata	Terrestrial snail	Metazoa-Mollusca-Gastropoda-Stylommatophora-Geomitridae-Candidula-unifasciata
Canis lupus_familiaris	Dog	Metazoa-Chordata-Mammalia-Carnivora-Canidae-Canis-lupus_familiaris
Capitella teleta	Marine worm	Metazoa-Annelida-Polychaeta-Order_unclassified-Capitellidae-Capitella-teleta
Capra hircus	Goat	Metazoa-Chordata-Mammalia-Artiodactyla-Bovidae-Capra-hircus
Capsaspora owczarzaki	Unicell	Kingdom_unclassified-Phylum_unclassified-Filasterea-Order_unclassified-Family_unclassified-Capsaspora-owczarzaki
Carassius auratus	Goldfish	Metazoa-Chordata-Actinopteri-Cypriniformes-Cyprinidae-Carassius-auratus
Carcharodon carcharias	Great white shark	Metazoa-Chordata-Chondrichthyes-Lamniformes-Alopiidae-Carcharodon-carcharias
Carlito syrichta	Philippine tarsier	Metazoa-Chordata-Mammalia-Primates-Tarsiidae-Carlito-syrichta
Cavia aperea	Brazilian guinea pig	Metazoa-Chordata-Mammalia-Rodentia-Caviidae-Cavia-aperea
Chiloscyllium plagiosum	White spotted bamboo shark	Metazoa-Chordata-Chondrichthyes-Orectolobiformes-Hemiscylliidae-Chiloscyllium-plagiosum

Chinchilla lanigera	Long-tailed chinchilla	Metazoa-Chordata-Mammalia-Rodentia-Chinchillidae-Chinchilla-lanigera
Choloepus didactylus	Xenarthra southern two-toed sloth	Metazoa-Chordata-Mammalia-Pilosa-Megalonychidae-Choloepus-didactylus
Chrysemys picta	Painted turtle	Metazoa-Chordata-Class_unclassified-Testudines-Emydidae-Chrysemys-picta
Chrysochloris asiatica	Cape golden mole	Metazoa-Chordata-Mammalia-Afrosoricida-Chrysochloridae-Chrysochloris-asiatica
Chrysomallon squamiferum	Sea pangolin	Metazoa-Mollusca-Gastropoda-Order_unclassified-Peltospiridae-Chrysomallon-squamiferum
Chrysoperla carnea	Green lacewing	Metazoa-Arthropoda-Insecta-Neuroptera-Chrysopidae-Chrysoperla-carnea
Ciona intestinalis	Vase tunicate	Metazoa-Chordata-Ascidiacea-Phlebobranchia-Cionidae-Ciona-intestinalis
Clarias magur	Walking catfish	Metazoa-Chordata-Actinopteri-Siluriformes-Clariidae-Clarias-magur
Cloeon dipterum	Mayfly	Metazoa-Arthropoda-Insecta-Ephemeroptera-Baetidae-Cloeon-dipterum
Clonorchis sinensis	Chinese liver fluke	Metazoa-Platyhelminthes-Trematoda-Opisthorchiida-Opisthorchiidae-Clonorchis-sinensis
Clytia hemisphaerica	Brady's hydrozoan	Metazoa-Cnidaria-Hydrozoa-Leptothecata-Clytiidae-Clytia-hemisphaerica
Coccinella septempunctata	Seven-spotted ladybird	Metazoa-Arthropoda-Insecta-Coleoptera-Coccinellidae-Coccinella-septempunctata
Colossoma macropomum	Black pacu	Metazoa-Chordata-Actinopteri-Characiformes-Serrasalmidae-Colossoma-macropomum
Condylura cristata	Star-nosed mole	Metazoa-Chordata-Mammalia-Eulipotyphla-Talpidae-Condylura-cristata
Conus betulinus	Cone snail	Metazoa-Mollusca-Gastropoda-Neogastropoda-Conidae-Conus-betulinus
Coptotermes formosanus	Formosan subterranean termite	Metazoa-Arthropoda-Insecta-Blattodea-Rhinotermitidae-Coptotermes-formosanus
Coregonus clupeaformis	Lake whitefish	Metazoa-Chordata-Actinopteri-Salmoniformes-Salmonidae-Coregonus-clupeaformis
Corvus moneduloides	New caledonian crow	Metazoa-Chordata-Aves-Passeriformes-Corvidae-Corvus-moneduloides
Crassostrea ariakensis	Suminoe oyster	Metazoa-Mollusca-Bivalvia-Ostreida-Ostreidae-Crassostrea-ariakensis
Crassostrea gigas	Pacific oyster	Metazoa-Mollusca-Bivalvia-Ostreida-Ostreidae-Crassostrea-gigas
Crassostrea virginica	Atlantic oyster	Metazoa-Mollusca-Bivalvia-Ostreida-Ostreidae-Crassostrea-virginica
Crocodylus porosus	Australian saltwater crocodile	Metazoa-Chordata-Class_unclassified-Crocodylia-Crocodylidae-Crocodylus-porosus
Crocuta crocuta	Spotted hyena	Metazoa-Chordata-Mammalia-Carnivora-Hyaenidae-Crocuta-crocuta
Cryptotermes secundus	Termite	Metazoa-Arthropoda-Insecta-Blattodea-Kalotermitidae-Cryptotermes-secundus
Ctenocephalides felis	Cat fleas	Metazoa-Arthropoda-Insecta-Siphonaptera-Pulicidae-Ctenocephalides-felis
Cyclina sinensis	Black clam	Metazoa-Mollusca-Bivalvia-Venerida-Veneridae-Cyclina-sinensis
Cyclopterus lumpus	Lumpsucker	Metazoa-Chordata-Actinopteri-Perciformes-Cyclopteridae-Cyclopterus-lumpus
Cyprinus carpio	Common carp	Metazoa-Chordata-Actinopteri-Cypriniformes-Cyprinidae-Cyprinus-carpio
Danaus plexippus	Monarch butterfly	Metazoa-Arthropoda-Insecta-Lepidoptera-Nymphalidae-Danaus-plexippus
Danio rerio	Zebrafish	Metazoa-Chordata-Actinopteri-Cypriniformes-Danionidae-Danio-rerio

Daphnia magna	Waterflea	Metazoa-Arthropoda-Branchiopoda-Diplostraca-Daphniidae-Daphnia-magna
Dasypus novemcinctus	Xenarthra nine-banded armadillo	Metazoa-Chordata-Mammalia-Cingulata-Dasypodidae-Dasypus-novemcinctus
Delphinapterus leucas	Beluga whale	Metazoa-Chordata-Mammalia-Artiodactyla-Monodontidae-Delphinapterus-leucas
Dicyema japonicum	Dicyemid	Metazoa-Dicyemida-Class_unclassified-Order_unclassified-Dicyemidae-Dicyema-japonicum
Dimorphilus gyrociliatus	Polychaete worm	Metazoa-Annelida-Polychaeta-Order_unclassified-Dinophilidae-Dimorphilus-gyrociliatus
Diploscapter coronatus	Nematode V	Metazoa-Nematoda-Chromadorea-Rhabditida-Rhabditidae-Diploscapter-coronatus
Ditylenchus destructor	Nematode IV	Metazoa-Nematoda-Chromadorea-Rhabditida-Anguinidae-Ditylenchus-destructor
Ditylenchus dipsaci	Nematode IV	Metazoa-Nematoda-Chromadorea-Rhabditida-Anguinidae-Ditylenchus-dipsaci
Dreissena polymorpha	Zebra mussel	Metazoa-Mollusca-Bivalvia-Myida-Dreissenidae-Dreissena-polymorpha
Dreissena rostriformis	Quagga mussel Dres	Metazoa-Mollusca-Bivalvia-Myida-Dreissenidae-Dreissena-rostriformis
Dromiciops gliroides	Marsupial Monito del monte	Metazoa-Chordata-Mammalia-Microbiotheria-Microbiotheriidae-Dromiciops-gliroides
Drosophila melanogaster	Fruit fly	Metazoa-Arthropoda-Insecta-Diptera-Drosophilidae-Drosophila-melanogaster
Echinococcus multilocularis	Tapeworm	Metazoa-Platyhelminthes-Cestoda-Cyclophyllidea-Taeniidae-Echinococcus-multilocularis
Echinops telfairi	Small Madagascar hedgehog	Metazoa-Chordata-Mammalia-Afrosoricida-Tenrecidae-Echinops-telfairi
Eisenia andrei	Tiger worm relative	Metazoa-Annelida-Clitellata-Crassiclitellata-Lumbricidae-Eisenia-andrei
Electrophorus electricus	Electric eel	Metazoa-Chordata-Actinopteri-Gymnotiformes-Gymnotidae-Electrophorus-electricus
Elephantulus edwardii	Cape elephant shrew	Metazoa-Chordata-Mammalia-Macroscelidea-Macroscelidea-Elephantulus-edwardii
Elysia chlorotica	Sea slug photosynthesis	Metazoa-Mollusca-Gastropoda-Order_unclassified-Plakobranchidae-Elysia-chlorotica
Elysia marginata	Sea slug photosynthesis	Metazoa-Mollusca-Gastropoda-Order_unclassified-Plakobranchidae-Elysia-marginata
Ephemera danica	Mayfly	Metazoa-Arthropoda-Insecta-Ephemeroptera-Ephemeridae-Ephemera-danica
Ephydatia muelleri	Sea2Water sponge	Metazoa-Porifera-Demospongiae-Spongillida-Spongillidae-Ephydatia-muelleri
Eptatretus burgeri	Inshore hagfish	Metazoa-Chordata-Myxini-Myxiniformes-Myxinidae-Eptatretus-burgeri
Equus caballus	Horse	Metazoa-Chordata-Mammalia-Perissodactyla-Equidae-Equus-caballus
Erinaceus europaeus	Western european hedgehog	Metazoa-Chordata-Mammalia-Eulipotyphla-Erinaceidae-Erinaceus-europaeus
Eulimnadia texana	Desert shrimp	Metazoa-Arthropoda-Branchiopoda-Diplostraca-Limnadiidae-Eulimnadia-texana
Eurytemora affinis	Copepod	Metazoa-Arthropoda-Hexanauplia-Calanoida-Temoridae-Eurytemora-affinis
Felis catus	Cat	Metazoa-Chordata-Mammalia-Carnivora-Felidae-Felis-catus
Folsomia candida	Springtail	Metazoa-Arthropoda-Collembola-Entomobryomorpha-Isotomidae-Folsomia-candida
Frankliniella occidentalis	Western flower thrips	Metazoa-Arthropoda-Insecta-Thysanoptera-Thripidae-Frankliniella-occidentalis
Fukomys damarensis	Damara mole rat	Metazoa-Chordata-Mammalia-Rodentia-Bathyergidae-Fukomys-damarensis

Fundulus heteroclitus	Mummichog killifish Guppies	Metazoa-Chordata-Actinopteri-Cyprinodontiformes-Fundulidae-Fundulus-heteroclitus
Gadus morhua	Cod	Metazoa-Chordata-Actinopteri-Gadiformes-Gadidae-Gadus-morhua
Galemys pyrenaicus	Pyrenean desman	Metazoa-Chordata-Mammalia-Eulipotyphla-Talpidae-Galemys-pyrenaicus
Galeopterus variegatus	Sunda flying lemur	Metazoa-Chordata-Mammalia-Dermoptera-Cynocephalidae-Galeopterus-variegatus
Galleria mellonella	Greater wax moth	Metazoa-Arthropoda-Insecta-Lepidoptera-Pyralidae-Galleria-mellonella
Gigantopelta aegis	Deep sea snail	Metazoa-Mollusca-Gastropoda-Order_unclassified-Peltospiridae-Gigantopelta-aegis
Globicephala melas	Long-finned pilot whale	Metazoa-Chordata-Mammalia-Artiodactyla-Delphinidae-Globicephala-melas
Gopherus evgoodei	Goodes thornscrub tortoise	Metazoa-Chordata-Class_unclassified-Testudines-Testudinidae-Gopherus-evgoodei
Haemonchus contortus	Nematode V	Metazoa-Nematoda-Chromadorea-Strongylida-Haemonchidae-Haemonchus-contortus
Haliotis rubra	Black liped abalone	Metazoa-Mollusca-Gastropoda-Lepetellida-Haliotidae-Haliotis-rubra
Haliotis rufescens	Red abalone	Metazoa-Mollusca-Gastropoda-Lepetellida-Haliotidae-Haliotis-rufescens
Helobdella robusta	Leech	Metazoa-Annelida-Clitellata-Rhynchobdellida-Glossiphoniidae-Helobdella-robusta
Heterocephalus glaber	African naked mole rat	Metazoa-Chordata-Mammalia-Rodentia-Bathyergidae-Heterocephalus-glaber
Hippopotamus amphibius	Hippo	Metazoa-Chordata-Mammalia-Artiodactyla-Hippopotamidae-Hippopotamus-amphibius
Hipposideros armiger	Rhinolophid Hipposideridae great roundleaf bat	Metazoa-Chordata-Mammalia-Chiroptera-Hipposideridae-Hipposideros-armiger
Hirudo medicinalis	Medicinal leech	Metazoa-Annelida-Clitellata-Hirudinida-Hirudinidae-Hirudo-medicinalis
Hofstenia miamia	Acoel flatworm	Metazoa-Xenacoelomorpha-Class_unclassified-Acoela-Hofsteniidae-Hofstenia-miamia
Homarus americanus	American lobster	Metazoa-Arthropoda-Malacostraca-Decapoda-Nephropidae-Homarus-americanus
Homo sapiens	Human	Metazoa-Chordata-Mammalia-Primates-Hominidae-Homo-sapiens
Hormiphora californensis	Ctenophore	Metazoa-Ctenophora-Tentaculata-Cydippida-Pleurobrachiidae-Hormiphora-californensis
Hydra viridissima	Green hydra	Metazoa-Cnidaria-Hydrozoa-Anthoathecata-Hydridae-Hydra-viridissima
Hydra vulgaris	Hydra	Metazoa-Cnidaria-Hydrozoa-Anthoathecata-Hydridae-Hydra-vulgaris
Hydrophis curtus	Shaw's sea snake	Metazoa-Chordata-Lepidosauria-Squamata-Hydrophiidae-Hydrophis-curtus
Hymenolepis microstoma	Rodent tapeworm	Metazoa-Platyhelminthes-Cestoda-Cyclophyllidea-Hymenolepididae-Hymenolepis-microstoma
Hyposmocoma kahamanoa	Oahu moth	Metazoa-Arthropoda-Insecta-Lepidoptera-Cosmopterigidae-Hyposmocoma-kahamanoa
Hypsibius dujardini	Tardigrade	Metazoa-Tardigrada-Eutardigrada-Parachela-Hypsibiidae-Hypsibius-dujardini
Ictalurus punctatus	Channel catfish	Metazoa-Chordata-Actinopteri-Siluriformes-Ictaluridae-Ictalurus-punctatus
Kryptolebias marmoratus	Mangrove rivulus killifish Guppies	Metazoa-Chordata-Actinopteri-Cyprinodontiformes-Rivulidae-Kryptolebias-marmoratus
Ladona fulva	Scarce chaser	Metazoa-Arthropoda-Insecta-Odonata-Libellulidae-Ladona-fulva
Lagenorhynchus obliquidens	Pacific white-sided dolphin	Metazoa-Chordata-Mammalia-Artiodactyla-Delphinidae-Lagenorhynchus-obliquidens

Lamellibrachia luymesi	Deep sea worm	Metazoa-Annelida-Class_unresolved-Order_unresolved-Family_unresolved-Lamellibrachia-luymsi
Laticauda laticaudata	Blue-ringed sea krait	Metazoa-Chordata-Lepidosauria-Squamata-Elapidae-Laticauda-laticaudata
Latimeria chalumnae	Coelacanth	Metazoa-Chordata-Class_unclassified-Coelacanthiformes-Coelacanthidae-Latimeria-chalumnae
Lautoconus ventricosus	Cone snail	Metazoa-Mollusca-Class_unresolved-Order_unresolved-Family_unresolved-Lautoconus-ventricosus
Lepas anserifera	Gooseneck barnacle	Metazoa-Arthropoda-Hexanauplia-Pedunculata-Lepadidae-Lepas-anserifera
Lepeoptheirus salmonis	Sea louse copepod	Metazoa-Arthropoda-Class_unresolved-Order_unresolved-Family_unresolved-Lepeoptheirus-salmonis
Lepidurus apus_apus	Tadpole shrimp	Metazoa-Arthropoda-Branchiopoda-Notostraca-Triopsidae-Lepidurus-apus_apus
Lepidurus apus_lubbocki	Tadpole shrimp	Metazoa-Arthropoda-Branchiopoda-Notostraca-Triopsidae-Lepidurus-apus_lubbocki
Lepidurus arcticus	Tadpole shrimp	Metazoa-Arthropoda-Branchiopoda-Notostraca-Triopsidae-Lepidurus-arcticus
Leptobrachium leishanense	Leishan spiny toad	Metazoa-Chordata-Amphibia-Anura-Megophryidae-Leptobrachium-leishanense
Limnoperna fortunei	Golden mussel	Metazoa-Mollusca-Bivalvia-Mytiloida-Mytilidae-Limnoperna-fortunei
Limulus polyphemus	Horseshoe crab	Metazoa-Arthropoda-Merostomata-Xiphosura-Limulidae-Limulus-polyphemus
Lingula anatina	Lampshell	Metazoa-Brachiopoda-Lingulata-Lingulida-Lingulidae-Lingula-anatina
Liparis tanakae	Tanaka snailfish	Metazoa-Chordata-Actinopteri-Perciformes-Liparidae-Liparis-tanakae
Lipotes vexillifer	Yangtze River dolphin	Metazoa-Chordata-Mammalia-Artiodactyla-Lipotidae-Lipotes-vexillifer
Lottia gigantea	Owl limpet	Metazoa-Mollusca-Gastropoda-Order_unclassified-Lottiidae-Lottia-gigantea
Loxodonta africana	Afrotherian african savanna elephant	Metazoa-Chordata-Mammalia-Proboscidea-Elephantidae-Loxodonta-africana
Lytechinus variegatus	Green sea urchin	Metazoa-Echinodermata-Echinoidea-Temnopleuroida-Toxopneustidae-Lytechinus-variegatus
Macrobrachium nipponense	Oriental river prawn	Metazoa-Arthropoda-Malacostraca-Decapoda-Palaemonidae-Macrobrachium-nipponense
Macrostomum lignano	Marine flatworm	Metazoa-Platyhelminthes-Rhabditophora-Macrostomida-Macrostomidae-Macrostomum-lignano
Manis pentadactyla	Chinese pangolin	Metazoa-Chordata-Mammalia-Pholidota-Manidae-Manis-pentadactyla
Margaritifera margaritifera	Sea2Water pearl mussel Unio	Metazoa-Mollusca-Bivalvia-Unionida-Margaritiferidae-Margaritifera-margaritifera
Marisa cornuarietis	Colombian ramshorn apple snail	Metazoa-Mollusca-Gastropoda-Architaenioglossa-Ampullariidae-Marisa-cornuarietis
Mercenaria mercenaria	Hard clam	Metazoa-Mollusca-Bivalvia-Venerida-Veneridae-Mercenaria-mercenaria
Meriones unguiculatus	Mongolian gerbil	Metazoa-Chordata-Mammalia-Rodentia-Muridae-Meriones-unguiculatus
Mesocricetus auratus	Golden hamster	Metazoa-Chordata-Mammalia-Rodentia-Cricetidae-Mesocricetus-auratus
Metaphire vulgaris	Earthworm	Metazoa-Annelida-Clitellata-Crassiclitellata-Megascolecidae-Metaphire-vulgaris
Microcebus murinus	Grey mouse lemur	Metazoa-Chordata-Mammalia-Primates-Cheirogaleidae-Microcebus-murinus
Miniopterus natalensis	Vespertilionoidid Miniopteridae Natal long-fingered bat	Metazoa-Chordata-Mammalia-Chiroptera-Vespertilionidae-Miniopterus-natalensis

Mizuhopecten yessoensis	Giant exo scallop	Metazoa-Mollusca-Bivalvia-Pectinida-Pectinidae-Mizuhopecten-yessoensis
Mnemiopsis leidyi	Warty comb jelly	Metazoa-Ctenophora-Tentaculata-Lobata-Bolinopsidae-Mnemiopsis-leidyi
Modiolus philippinarum	Philippine horse mussel	Metazoa-Mollusca-Bivalvia-Mytiloida-Mytilidae-Modiolus-philippinarum
Molossus molossus	Vespertilionoidid Molossidae Pallas's mastiff bat	Metazoa-Chordata-Mammalia-Chiroptera-Molossidae-Molossus-molossus
Monodelphis domestica	Marsupial Grey short-tailed opposum	Metazoa-Chordata-Mammalia-Didelphimorphia-Didelphidae-Monodelphis-domestica
Monodon monoceros	Narwhal	Metazoa-Chordata-Mammalia-Artiodactyla-Monodontidae-Monodon-monoceros
Monosiga brevicollis	Unicellular choanoflagellate	Kingdom_unclassified-Phylum_unclassified-Choanoflagellata-Craspedida-Salpingoecidae-Monosiga-brevicollis
Morbakka virulenta	Box jellyfish	Metazoa-Cnidaria-Cubozoa-Carybdeida-Carukiidae-Morbakka-virulenta
Mus musculus	Mouse	Metazoa-Chordata-Mammalia-Rodentia-Muridae-Mus-musculus
Mytilus coruscus	Korean mussel	Metazoa-Mollusca-Bivalvia-Mytiloida-Mytilidae-Mytilus-coruscus
Mytilus edulis	Blue mussel	Metazoa-Mollusca-Bivalvia-Mytiloida-Mytilidae-Mytilus-edulis
Mytilus galloprovincialis	Mediterraean mussel	Metazoa-Mollusca-Bivalvia-Mytiloida-Mytilidae-Mytilus-galloprovincialis
Naja naja	Indian cobra	Metazoa-Chordata-Lepidosauria-Squamata-Elapidae-Naja-naja
Nannospalax galili	Upper Galilee mountains blind mole rat	Metazoa-Chordata-Mammalia-Rodentia-Spalacidae-Nannospalax-galili
Nautilus pompilius	Nautilus	Metazoa-Mollusca-Cephalopoda-Nautilida-Nautilidae-Nautilus-pompilius
Nematostella vectensis	Starlet sea anemone	Metazoa-Cnidaria-Anthozoa-Actiniaria-Edwardsiidae-Nematostella-vectensis
Neoceratodus forsteri	Australian lungfish	Metazoa-Chordata-Class_unclassified-Ceratodontiformes-Ceratodontidae-Neoceratodus-forsteri
Neophocaena asiaeorientalis_asiaeorientalis	Yangtze finless porpoise	Metazoa-Chordata-Mammalia-Artiodactyla-Phocoenidae-Neophocaena-asiaeorientalis_asiaeorientalis
Nilaparvata lugens	Brown planthopper	Metazoa-Arthropoda-Insecta-Hemiptera-Delphacidae-Nilaparvata-lugens
Notechis scutatus	Mainland tiger snake	Metazoa-Chordata-Lepidosauria-Squamata-Elapidae-Notechis-scutatus
Nothobranchius furzeri	Torquoise killifish Guppies	Metazoa-Chordata-Actinopteri-Cyprinodontiformes-Nothobranchiidae-Nothobranchius-furzeri
Notospermus geniculatus	Nemertean worm	Metazoa-Nemertea-Pilidiophora-Heteronemertea-Lineidae-Notospermus-geniculatus
Nymphon striatum	Sea spider	Metazoa-Arthropoda-Pycnogonida-Pantopoda-Nymphonidae-Nymphon-striatum
Octodon degus	Degu	Metazoa-Chordata-Mammalia-Rodentia-Octodontidae-Octodon-degus
Octopus sinensis	East asian common octopus	Metazoa-Mollusca-Cephalopoda-Octopoda-Octopodidae-Octopus-sinensis
Oikopleura dioica	Pelagic seasquirt	Metazoa-Chordata-Appendicularia-Copelata-Oikopleuridae-Oikopleura-dioica
Onchocerca volvulus	Nematode III	Metazoa-Nematoda-Chromadorea-Rhabditida-Onchocercidae-Onchocerca-volvulus
Oncorhynchus mykiss	Rainbow trout	Metazoa-Chordata-Actinopteri-Salmoniformes-Salmonidae-Oncorhynchus-mykiss
Orcinus orca	Killer whale	Metazoa-Chordata-Mammalia-Artiodactyla-Delphinidae-Orcinus-orca

Ornithorhynchus anatinus	Montreme platypus	Metazoa-Chordata-Mammalia-Monotremata-Ornithorhynchidae-Ornithorhynchus-anatinus
Orycteropus afer_afer	Afrotherian aardvark	Metazoa-Chordata-Mammalia-Tubulidentata-Orycteropodidae-Orycteropus-afer_afer
Oryctolagus cuniculus	Lagomorphan rabbit	Metazoa-Chordata-Mammalia-Lagomorpha-Leporidae-Oryctolagus-cuniculus
Oryzias latipes	Medaka Guppies	Metazoa-Chordata-Actinopteri-Beloniformes-Adrianichthyidae-Oryzias-latipes
Owenia fusiformis	Owenid tubeworm	Metazoa-Annelida-Polychaeta-Sabellida-Oweniidae-Owenia-fusiformis
Panagrolaimus es5	Panagrolaimus nematode IV	Metazoa-Nematoda-Class_unresolved-Order_unresolved-Family_unresolved-Panagrolaimus-es5
Panagrolaimus ps1159	Panagrolaimus nematode IV	Metazoa-Nematoda-Class_unresolved-Order_unresolved-Family_unresolved-Panagrolaimus-ps1159
Panagrolaimus superbus	Panagrolaimus nematode IV	Metazoa-Nematoda-Chromadorea-Rhabditida-Panagrolaimidae-Panagrolaimus-superbus
Pangasianodon hypophthalmus	Iridescent shark catfish	Metazoa-Chordata-Actinopteri-Siluriformes-Pangasiidae-Pangasianodon-hypophthalmus
Papilio xuthus	Asian swallowtail butterfly	Metazoa-Arthropoda-Insecta-Lepidoptera-Papilionidae-Papilio-xuthus
Paraescarpia echinospica	Vestimentiferan tubeworm	Metazoa-Annelida-Polychaeta-Sabellida-Siboglinidae-Paraescarpia-echinospica
Paramormyrops kingsleyae	Elephantfish electric fish	Metazoa-Chordata-Actinopteri-Osteoglossiformes-Mormyridae-Paramormyrops-kingsleyae
Pararge aegeria	Specked wood butterfly	Metazoa-Arthropoda-Insecta-Lepidoptera-Nymphalidae-Pararge-aegeria
Patiria miniata	Bat star	Metazoa-Echinodermata-Asteroidea-Valvatida-Asterinidae-Patiria-miniata
Pecten maximus	Great scallop	Metazoa-Mollusca-Bivalvia-Pectinida-Pectinidae-Pecten-maximus
Pediculus humanus_corporis	Human louse	Metazoa-Arthropoda-Insecta-Phthiraptera-Pediculidae-Pediculus-humanus_corporis
Penaeus monodon	Giant tiger prawn	Metazoa-Arthropoda-Malacostraca-Decapoda-Penaeidae-Penaeus-monodon
Periophthalmus magnuspinnatus	Mudskipper Gobies	Metazoa-Chordata-Actinopteri-Gobiiformes-Gobiidae-Periophthalmus-magnuspinnatus
Perna viridis	Asian green mussel	Metazoa-Mollusca-Bivalvia-Mytiloida-Mytilidae-Perna-viridis
Petromyzon marinus	Inshore lamprey	Metazoa-Chordata-Hyperoartia-Petromyzontiformes-Petromyzontidae-Petromyzon-marinus
Phascolarctos cinereus	Marsupial Koala	Metazoa-Chordata-Mammalia-Diprotodontia-Phascolarctidae-Phascolarctos-cinereus
Phoca vitulina	Harbor seal	Metazoa-Chordata-Mammalia-Carnivora-Phocidae-Phoca-vitulina
Phocoena sinus	Vaquita	Metazoa-Chordata-Mammalia-Artiodactyla-Phocoenidae-Phocoena-sinus
Phoronis australis	Phoronid worm	Metazoa-Phoronida-Class_unclassified-Order_unclassified-Family_unclassified-Phoronis-australis
Photinus pyralis	Common eastern firefly	Metazoa-Arthropoda-Insecta-Coleoptera-Lampyridae-Photinus-pyralis
Phyllostomus discolor	Noctilionoidid Phyllostomidae pale spear-nosed bat	Metazoa-Chordata-Mammalia-Chiroptera-Phyllostomidae-Phyllostomus-discolor
Physeter catodon	Sperm whale	Metazoa-Chordata-Mammalia-Artiodactyla-Physeteridae-Physeter-catodon
Pinctada fucata	Akoya pearl oyster	Metazoa-Mollusca-Bivalvia-Pterioida-Pteriidae-Pinctada-fucata

Pipistrellus kuhlii	Vespertilionoidid Vespertilionidae Kuhl's pipistrelle	Metazoa-Chordata-Mammalia-Chiroptera-Vespertilionidae-Pipistrellus-kuhlii
Plakobranchus ocellatus	Sea slug	Metazoa-Mollusca-Gastropoda-Order_unclassified-Plakobranchidae-Plakobranchus-ocellatus
Plectus sambesii	Nematode C	Metazoa-Nematoda-Chromadorea-Plectida-Plectidae-Plectus-sambesii
Plutella xylostella	Diamondback moth	Metazoa-Arthropoda-Insecta-Lepidoptera-Plutellidae-Plutella-xylostella
Poecilia latipinna	Molly Guppies	Metazoa-Chordata-Actinopteri-Cyprinodontiformes-Poeciliidae-Poecilia-latipinna
Poeciliopsis occidentalis	Topminnow Guppies	Metazoa-Chordata-Actinopteri-Cyprinodontiformes-Poeciliidae-Poeciliopsis-occidentalis
Polistes fuscatus	common paper wasp	Metazoa-Arthropoda-Insecta-Hymenoptera-Vespidae-Polistes-fuscatus
Pollicipes pollicipes	Barnacle	Metazoa-Arthropoda-Hexanauplia-Pedunculata-Pollicipedidae-Pollicipes-pollicipes
Polypedilum vanderplanki	Sleeping chironomid midge	Metazoa-Arthropoda-Insecta-Diptera-Chironomidae-Polypedilum-vanderplanki
Pomacea canaliculata	Golden apple snail	Metazoa-Mollusca-Gastropoda-Architaenioglossa-Ampullariidae-Pomacea-canaliculata
Pontoporia blainvillei	Franciscana	Metazoa-Chordata-Mammalia-Artiodactyla-Pontoporiidae-Pontoporia-blainvillei
Portunus trituberculatus	Swimming crab	Metazoa-Arthropoda-Malacostraca-Decapoda-Portunidae-Portunus-trituberculatus
Potamilus streckersoni	Sea2Water mussel Unio	Metazoa-Mollusca-Bivalvia-Unionida-Unionidae-Potamilus-streckersoni
Priapulus caudatus	Priapulid worm	Metazoa-Priapulida-Priapulimorpha-Priapulimorphida-Priapulidae-Priapulus-caudatus
Pristionchus pacificus	Genetic model nematode V	Metazoa-Nematoda-Chromadorea-Rhabditida-Neodiplogasteridae-Pristionchus-pacificus
Procambarus virginalis	Crayfish	Metazoa-Arthropoda-Malacostraca-Decapoda-Cambaridae-Procambarus-virginalis
Propanagrolaimus ju765	Propanagrolaimus nematode IV	Metazoa-Nematoda-Class_unresolved-Order_unresolved-Family_unresolved-Propanagrolaimus-ju765
Protopterus annectens	African lungfish	Metazoa-Chordata-Class_unclassified-Ceratodontiformes-Protopteridae-Protopterus-annectens
Pseudoliparis swirei	Mariana hadal snailfish	Metazoa-Chordata-Actinopteri-Perciformes-Liparidae-Pseudoliparis-swirei
Pseudoliparis YAP	Hadal snailfish	Metazoa-Chordata-Class_unresolved-Order_unresolved-Family_unresolved-Pseudoliparis-YAP
Pseudonaja textilis	Eastern brown snake	Metazoa-Chordata-Lepidosauria-Squamata-Elapidae-Pseudonaja-textilis
Pygocentrus nattereri	Piranha	Metazoa-Chordata-Actinopteri-Characiformes-Serrasalmidae-Pygocentrus-nattereri
Ramazzottius varieornatus	Tardigrade	Metazoa-Tardigrada-Eutardigrada-Parachela-Ramazzottiidae-Ramazzottius-varieornatus
Rana temporaria	Frog	Metazoa-Chordata-Amphibia-Anura-Ranidae-Rana-temporaria
Rattus norvegicus	Rat	Metazoa-Chordata-Mammalia-Rodentia-Muridae-Rattus-norvegicus
Rhinolophus ferrumequinum	Rhinolophid Rhinolophidae greater horseshoe bat	Metazoa-Chordata-Mammalia-Chiroptera-Rhinolophidae-Rhinolophus-ferrumequinum
Rhizomys pruinosus	Hoary bamboo rat	Metazoa-Chordata-Mammalia-Rodentia-Spalacidae-Rhizomys-pruinosus
Rhopalosiphum maidis	Corn leaf aphid	Metazoa-Arthropoda-Insecta-Hemiptera-Aphididae-Rhopalosiphum-maidis
Rhopilema esculentum	Flame jellyfish	Metazoa-Cnidaria-Scyphozoa-Rhizostomeae-Rhizostomatidae-Rhopilema-esculentum

Rotaria socialis	Rotifer	Metazoa-Rotifera-Eurotatoria-Order_unclassified-Family_unclassified-Rotaria-socialis
Rousettus aegyptiacus	Pteropodid Pteropodidae egyptian rousette	Metazoa-Chordata-Mammalia-Chiroptera-Pteropodidae-Rousettus-aegyptiacus
Saccoglossus kowalevskii	Acorn worm	Metazoa-Hemichordata-Enteropneusta-Order_unclassified-Harrimaniidae-Saccoglossus-kowalevskii
Saccostrea glomerata	Sydney rock oyster	Metazoa-Mollusca-Bivalvia-Ostreida-Ostreidae-Saccostrea-glomerata
Salmo salar	Salmon	Metazoa-Chordata-Actinopteri-Salmoniformes-Salmonidae-Salmo-salar
Salvelinus namaycush	Lake trout	Metazoa-Chordata-Actinopteri-Salmoniformes-Salmonidae-Salvelinus-namaycush
Sarcophilus harrisii	Marsupial Tasmanian devil	Metazoa-Chordata-Mammalia-Dasyuromorphia-Dasyuridae-Sarcophilus-harrisii
Scapharca broughtonii	Blood clam	Metazoa-Mollusca-Class_unresolved-Order_unresolved-Family_unresolved-Scapharca-broughtonii
Schistosoma mansoni	Blood fluke	Metazoa-Platyhelminthes-Trematoda-Strigeidida-Schistosomatidae-Schistosoma-mansoni
Schmidtea mediterranea	Sea2Water triclad flatworm	Metazoa-Platyhelminthes-Rhabditophora-Tricladida-Dugesiidae-Schmidtea-mediterranea
Scleropages formosus	Asian arowana electric fish	Metazoa-Chordata-Actinopteri-Osteoglossiformes-Osteoglossidae-Scleropages-formosus
Sepia pharaonis	Pharaoh cuttlefish	Metazoa-Mollusca-Cephalopoda-Sepiida-Sepiidae-Sepia-pharaonis
Sinocyclocheilus anshuiensis	Barbine	Metazoa-Chordata-Actinopteri-Cypriniformes-Cyprinidae-Sinocyclocheilus-anshuiensis
Sinocyclocheilus grahami	Golden barbel barbine	Metazoa-Chordata-Actinopteri-Cypriniformes-Cyprinidae-Sinocyclocheilus-grahami
Sinocyclocheilus rhinocerous	Barbine	Metazoa-Chordata-Actinopteri-Cypriniformes-Cyprinidae-Sinocyclocheilus-rhinocerous
Sinonovacula constricta	Razor clam	Metazoa-Mollusca-Bivalvia-Cardiida-Solecurtidae-Sinonovacula-constricta
Solenopsis invicta	Fire ant	Metazoa-Arthropoda-Insecta-Hymenoptera-Formicidae-Solenopsis-invicta
Sorex araneus	European shrew	Metazoa-Chordata-Mammalia-Eulipotyphla-Soricidae-Sorex-araneus
Sousa chinensis	Indo-pacific humpbacked dolphin	Metazoa-Chordata-Mammalia-Artiodactyla-Delphinidae-Sousa-chinensis
Sphaeroforma arctica	Unicell	Kingdom_unclassified-Phylum_unclassified-Ichthyosporea-Ichthyophonida-Family_unclassified-Sphaeroforma-arctica
Sphenodon punctatus	Tuatara	Metazoa-Chordata-Lepidosauria-Sphenodontia-Sphenodontidae-Sphenodon-punctatus
Spodoptera frugiperda	Fall armyworm moth	Metazoa-Arthropoda-Insecta-Lepidoptera-Noctuidae-Spodoptera-frugiperda
Streblospio benedicti	Ram's horn worm	Metazoa-Annelida-Polychaeta-Spionida-Spionidae-Streblospio-benedicti
Strix occidentalis_caurina	Northern spotted owl	Metazoa-Chordata-Aves-Strigiformes-Strigidae-Strix-occidentalis_caurina
Strongylocentrotus purpuratus	Sea urchin	Metazoa-Echinodermata-Echinoidea-Camarodonta-Strongylocentrotidae-Strongylocentrotus-purpuratus
Styela clava	Stalked sea squirt	Metazoa-Chordata-Ascidiacea-Stolidobranchia-Styelidae-Styela-clava
Sus scrofa	Pig	Metazoa-Chordata-Mammalia-Artiodactyla-Suidae-Sus-scrofa
Taenia multiceps	Tapeworm	Metazoa-Platyhelminthes-Cestoda-Cyclophyllidea-Taeniidae-Taenia-multiceps
Taeniopygia guttata	Zebra finch	Metazoa-Chordata-Aves-Passeriformes-Estrildidae-Taeniopygia-guttata

Talpa occidentalis	Iberian mole	Metazoa-Chordata-Class_unresolved-Order_unresolved-Family_unresolved-Talpa-occidentali
Teleogryllus occipitalis	Cricket	Metazoa-Arthropoda-Insecta-Orthoptera-Gryllidae-Teleogryllus-occipitalis
Tetraodon nigroviridis	Pufferfish	Metazoa-Chordata-Actinopteri-Tetraodontiformes-Tetraodontidae-Tetraodon-nigroviridis
Thelohanellus kitauei	Mxyozoan parasite	Metazoa-Cnidaria-Myxozoa-Bivalvulida-Myxobolidae-Thelohanellus-kitauei
Thrips palmi	Thrip	Metazoa-Arthropoda-Insecta-Thysanoptera-Thripidae-Thrips-palmi
Tigriopus californicus	Tigger Pod copepod	Metazoa-Arthropoda-Hexanauplia-Harpacticoida-Harpacticidae-Tigriopus-californicus
Tribolium madens	Black flour beetle	Metazoa-Arthropoda-Insecta-Coleoptera-Tenebrionidae-Tribolium-madens
Trichechus manatus_latirostris	Florida manatee	Metazoa-Chordata-Mammalia-Sirenia-Trichechidae-Trichechus-manatus_latirostris
Trichoplax adhaerens	Placozoan	Metazoa-Placozoa-Class_unclassified-Order_unclassified-Trichoplacidae-Trichoplax-adhaerens
Triops cancriformis	Tadpole shrimp	Metazoa-Arthropoda-Branchiopoda-Notostraca-Triopsidae-Triops-cancriformis
Triops longicaudatus	Tadpole shrimp	Metazoa-Arthropoda-Branchiopoda-Notostraca-Triopsidae-Triops-longicaudatus
Tupaia chinensis	Scandentian chinese tree shrew	Metazoa-Chordata-Mammalia-Scandentia-Tupaiidae-Tupaia-chinensis
Tursiops truncatus	Common bottlenose dolphin	Metazoa-Chordata-Mammalia-Artiodactyla-Delphinidae-Tursiops-truncatus
Vicugna vicugna_mensalis	Vicugna	Metazoa-Chordata-Mammalia-Artiodactyla-Camelidae-Vicugna-vicugna_mensalis
Vombatus ursinus	Marsupial Common wombat	Metazoa-Chordata-Mammalia-Diprotodontia-Vombatidae-Vombatus-ursinus
Xenopus laevis	African clawed frog	Metazoa-Chordata-Amphibia-Anura-Pipidae-Xenopus-laevis
Xenopus tropicalis	Western clawed frog	Metazoa-Chordata-Amphibia-Anura-Pipidae-Xenopus-tropicalis
Zalophus californianus	Sea lion	Metazoa-Chordata-Mammalia-Carnivora-Otariidae-Zalophus-californianus
Zerene cesonia	Dogface butterfly	Metazoa-Arthropoda-Insecta-Lepidoptera-Pieridae-Zerene-cesonia
Zootoca vivipara	Common lizard	Metazoa-Chordata-Lepidosauria-Squamata-Lacertidae-Zootoca-vivipara

Supplementary Table S2 – Species selected for genome-scale phylogenetic analysis of P2XRs.

Species used in the creation of figure 7 and supplementary figure S4 and the source for each genome.

Organism	Location of genome data
Dictyostelium discoideum	https://www.ncbi.nlm.nih.gov/data-hub/genome/GCF_000004695.1/
Monosiga brevicollis	https://www.ncbi.nlm.nih.gov/genome/713?genome_assembly_id=30632
Amphimedon queenslandica	https://www.ncbi.nlm.nih.gov/genome/annotation_euk/Amphimedon_queenslandica/102/
Mnemiopsis leidyi	https://metazoa.ensembl.org/Mnemiopsis_leidyi/Info/Annotation/#assembly
Trichoplax adhaerens	https://www.ncbi.nlm.nih.gov/assembly/GCF_000150275.1/#/st
Thelohanellus kitauei	https://www.ncbi.nlm.nih.gov/data-hub/genome/GCA_000827895.1/
Morbakka virulenta	https://marinegenomics.oist.jp/morbakka_virulenta/download/MOR05_r06_proteins.fa.gz
Nematostella vectensis	https://genomes.stowers.org/files/pub/nematostella/Nvec/genomes/Nvec200/aligned/tcs_v2/tcs_v2.20211013.protein.versioned.fasta
Acropora millepora	https://www.ncbi.nlm.nih.gov/genome/annotation_euk/Acropora_millepora/100/
Rhopilema esculentum	https://www.ncbi.nlm.nih.gov/data-hub/genome/GCA_013076305.1/
Clytia hemisphaerica	https://ftp.ensemblgenomes.ebi.ac.uk/pub/metazoa/release-56/fasta/clytia_hemisphaerica_gca902728285/pep/Clytia_hemisphaerica_gca902728285.GCA902728285v1.pep.all.fa.gz
Lottia gigantea	https://metazoa.ensembl.org/Lottia_gigantea/Info/Annotation/
Limulus polyphemus	https://www.ncbi.nlm.nih.gov/genome/annotation_euk/Limulus_polyphemus/101/
Branchiostoma floridae	https://www.ncbi.nlm.nih.gov/genome/annotation_euk/Branchiostoma_floridae/100/
Homo sapiens	https://www.ensembl.org/Homo_sapiens/Info/Annotation
Allomyces macrogynus	https://www.ncbi.nlm.nih.gov/data-hub/genome/GCA_000151295.1/
Ostreococcus tauri	https://www.ncbi.nlm.nih.gov/data-hub/genome/GCF_000214015.3/

Supplementary Table S3 – *Clytia* P2XR gene information

Table of the putative P2X gene locations within the Clytia genome.

Clytia name	Transcript	gene	coordinates	
ChP2X1	TCONS_00012680	XLOC_007123	sc0000147:234352..239148 +	
ChP2X2	TCONS_00058261	XLOC_036701	scaffold_309:662166..672147 +	
ChP2X3	TCONS_00058302	XLOC_036723	scaffold_309:672814..694604 -	
ChP2X4	TCONS_00058308	XLOC_036725	scaffold_309:695613..708230 -	

Supplementary References

S1. Sievers, F. *et al.* Fast, scalable generation of high-quality protein multiple sequence alignments using Clustal Omega. *Molecular Systems Biology* **7**, 539 (2011).
